# Dynamic magnetic resonance imaging of muscle contraction in facioscapulohumeral muscular dystrophy

**DOI:** 10.1038/s41598-022-11147-2

**Published:** 2022-05-04

**Authors:** Xeni Deligianni, Francesco Santini, Matteo Paoletti, Francesca Solazzo, Niels Bergsland, Giovanni Savini, Arianna Faggioli, Giancarlo Germani, Mauro Monforte, Enzo Ricci, Giorgio Tasca, Anna Pichiecchio

**Affiliations:** 1 Radiology, Division of Radiological Physics, University Hospital of Basel, Petersgraben 4, 4031 Basel, Switzerland; 2 Basel Muscle MRI, Department of Biomedical Engineering, University of Basel, Allschwil, Switzerland; 3 Neuroradiology Department, Advanced Imaging and Radiomics Center, IRCCS Mondino Foundation, Pavia, Italy; 4 School of Specialization in Clinical Pharmacology and Toxicology Center of Research in Medical Pharmacology, School of Medicine, University of Insubria, Varese, Italy; 5 Department of Neurology, Buffalo Neuroimaging Analysis Center, Jacobs School of Medicine and Biomedical Sciences, Buffalo, NY USA; 6 IRCCS Fondazione Don Carlo Gnocchi ONLUS, Milan, Italy; 7 Unità Operativa Complessa di Neurologia, Fondazione Policlinico Universitario A. Gemelli IRCCS, Rome, Italy; 8 Department of Brain and Behavioral Sciences, University of Pavia, Pavia, Italy

**Keywords:** Magnetic resonance imaging, Biomarkers, Neuromuscular disease

## Abstract

Quantitative muscle MRI (water-T2 and fat mapping) is being increasingly used to assess disease involvement in muscle disorders, while imaging techniques for assessment of the dynamic and elastic muscle properties have not yet been translated into clinics. In this exploratory study, we quantitatively characterized muscle deformation (strain) in patients affected by facioscapulohumeral muscular dystrophy (FSHD), a prevalent muscular dystrophy, by applying dynamic MRI synchronized with neuromuscular electrical stimulation (NMES). We evaluated the quadriceps muscles in 34 ambulatory patients and 13 healthy controls, at 6-to 12-month time intervals. While a subgroup of patients behaved similarly to controls, for another subgroup the median strain decreased over time (approximately 57% over 1.5 years). Dynamic MRI parameters did not correlate with quantitative MRI. Our results suggest that the evaluation of muscle contraction by NMES-MRI is feasible and could potentially be used to explore the elastic properties and monitor muscle involvement in FSHD and other neuromuscular disorders.

## Introduction

Facioscapulohumeral muscular dystrophy (FSHD) is a genetic disorder and one of the most prevalent muscular dystrophies^1,2^. In muscular dystrophies, skeletal muscle undergoes several pathophysiological processes encompassing necrosis, inflammation, fibrosis, and finally replacement by adipose tissue. The progression of muscle wasting and weakness in FSHD is peculiarly stepwise^3,4^, asymmetric^5,6^ and generally slow. However, the clinical phenotype of the disease can sometimes overlap with other types of muscle disorders^7^, and magnetic resonance imaging (MRI) can identify patterns of involvement that help with the differential diagnosis^3^.

Therefore, MRI in the last years has been increasingly applied to evaluate the extent of muscle involvement and evolution in FSHD^3,8–10^. Overall, there is strong evidence pointing to the direction that hyperintensities on short tau inversion recovery (STIR) images reflect an active phase of disease^3^, which is followed by fat infiltration^11,12^ typically identified on T1 weighted (T1w) images, as the disease progresses. Indeed, the results of several studies suggested that muscles presenting with STIR hyperintensity have a faster progression towards fat replacement^5,13^.

Functional measurements such as the 6-min walking test (6MWT)^14^ or force measurements^15^ are also relevant in the follow-up of neuromuscular patients, and for FSHD as well. Recently, it was observed that changes of fat fraction in FSHD occur more at a single muscle level, and the larger these changes are, the faster the change in muscle strength^12^.

However, while existing MRI techniques are able to identify fat replacement and areas of muscle necrosis/inflammation, they may not always be sufficiently sensitive to change^16^. In FSHD specifically, given the slow progression of the disease, even fat fraction changes might be hard to detect^12^ and therefore capturing disease progression in the short time frame of a clinical trial can be challenging. In addition, the deposition of collagen tissue, accounting for fibrosis and supposed to affect the elastic properties of skeletal muscle, is currently escaping detection by MRI.

Neuromuscular electrical stimulation (NMES)-synchronized MRI of muscle deformation has been used to characterize how muscles contract^17–19^ and provides functional information. This method gives an estimate of the contraction velocity as well as the deformation of muscles with strain maps. In a previous study using NMES-synchronized MRI, the strain from phase-contrast MRI was shown to be different between young and senior (> 70-year old) healthy volunteers^19^. Moreover, the rates at which the strain reaches the maximum (*build-up constant*)^19^ and relaxes to zero (*release constant)* can offer additional information about the ability of the muscle to deform. A healthier muscle is expected to have a faster build-up of strain, higher maximum, and faster release in electromyography-based assessments^20^. However, to our knowledge, there is no study to date on the application of similar dynamic MRI methods, either with evoked NMES or voluntary contraction, in patients with neuromuscular diseases. Nevertheless, NMES has been applied for training of muscular dystrophy patients, such as for example in case of FSHD^21^.

The purpose of this preliminary study was to characterize the muscle deformation behavior in FSHD patients compared to healthy controls followed longitudinally. For this aim, NMES was applied for the periodic contraction of the quadriceps muscle in synchronization with phase-contrast MRI to acquire dynamic data. Finally, the results from the dynamic analysis were compared to water T2 relaxation and fat fraction measurements.

## Methods

Ambulatory FSHD patients, who had a confirmed molecular diagnosis and were already enrolled in a larger study on longitudinal MRI biomarkers, were recruited^22^. Due to the length of the study, the healthy control subjects were selected amongst the persons accompanying the FSHD subjects, mainly between non-consanguineous ones (i.e., spouses and friends). The age and the weight of the control subjects were comparable to that of FSHD subjects. All healthy controls had no medical history suggestive of disorders that could impair muscle function and physical examination was normal. All experimental protocols and methods were approved by the local institutional review board regulations (by the “Comitato Etico Area Referente Pavia Fondazione IRCCS Policlinico San Matteo” at the Mondino Foundation and by the Ethics Committee-Prot. 7451/18 ID 1952 at the Policlinico Gemelli) and performed in accordance with the relevant guidelines and regulations. All volunteers signed an informed consent. Patients were clinically evaluated at baseline using the Clinical Severity Score (CSS)^23^, a specific score for FSHD ranging from 0 (asymptomatic patient) to 5 (non-ambulant patient), the 6MWT and the dynamometric evaluation (microFET® handheld dynamometer [Hoggan Scientific]) of the maximum voluntary contraction of the quadriceps. The length of the 4q35 BlnI resistant, p13-E11 EcoRI fragment associated with the disease was also reported (in kb).

All patients were examined at up to four time points: the first three 6 months apart (t0, t1, t2) and the last (t3), when available, after 12 months.Healthy controls (HCs) were scanned up to three times (at 6 to 12 month intervals).

The patients (12 female, 22 male) at the time point of the first scan were 44.9 ± 8.9 years old with weight 76.9 ± 19.5 kg and height 173.2 ± 9.3 cm (i.e., average weight and height of all datasets over all 3 time points). The healthy controls (HCs) (9 female, 4 male), were 48.5 ± 12.6 years old with weight 69.3 ± 11.3 kg and height 167.8 ± 7.5 cm.

Dynamic scans were performed for both thighs separately (i.e., two dynamic datasets per participant per time point). The quadriceps muscle was chosen due to its size and position that are ideal for NMES-MRI.

### Imaging/stimulation

For the dynamic MRI acquisition, a commercial NMES device (InTENSity Twin Stim III TENS and EMS Combo [Current Solutions LLC]) was synchronized with the MRI acquisition. Pairs of 5.1 × 8.9 cm^2^ gel-based NMES electrodes [TensUnits.com]) were employed for stimulation and exact measures of their position were taken for subsequent visits (i.e., distance between electrodes, distance from patella, lateral position on MRI). The median distance of the electrodes for both legs for FSHD patients was 11.0 cm (1st Quartile (Q1), 3rd Quartile (Q3) = 10,14) and for HCs 10.5 (Q1, Q3 = 10.0,12.38). A glycerol marker was positioned on each electrode for localization on the MRI. The electrodes were placed by identifying the motor point with a stimulation pen^24^. A training NMES session was performed for familiarization and the current was set to a sufficient level to evoke isometric contraction and without discomfort. For every subsequent scan, the current was set at the same level as previously, provided it evoked a similar visible muscle contraction, or otherwise higher^19^. When the volunteer reported discomfort or local pain, the current was lowered.

All subjects were scanned on a 3 T clinical MRI scanner (MAGNETOM Skyra [Siemens Healthineers]) with an 18-element body-array centered on the thigh. A three-directional single-slice cine phase-contrast (PC) velocity encoding acquisition was used in triggered mode during periodic contractions and a parasagittal slice was acquired^17,19^ (see Table 1). One channel of the NMES device was connected to the electrodes, while the other was used to simultaneously trigger the MRI acquisition^17,19^. The dynamic scans were performed as last. Each contraction cycle lasted 5 s (1 s ramp-up/down, 1 s contraction, 2 s relaxation).

**Table 1:** MRI parameters for dynamic and quantitative MRI.

	Dynamic scan	T2-mapping	Fat fraction mapping
	Single slice PC	2D multi-echo spin-echo (MESE)	Multi-echo gradient-echo (MEGE)
Voxel	2.3 × 2.3 × 5 mm^3^	1.2 × 1.2 × 10.0 mm^3^	1.0 × 1.0 × 5.0 mm^3^
Flip angle	10°	90°/ 180°	7°
Field-of-view	225 × 300 mm^2^	448 × 224	448 × 260
Bandwidth per pixel	400 Hz/px	250 Hz/px	1050 Hz/px
Repetition time (TR)/echo time (TE)	(TR/TE) = 10.6/7.21 ms	8–17 echoes, TR: 4100.0 ms, 1st TE & ΔTE: 10.9 ms	6 echoes, TR 35.0 ms, first TE/echo spacing 1.7/1.5 ms
Acquisition time	5 min	5 min	15 min
Other	Velocity encoding: 25 cm/s Temporal resolution: 42 ms, 1 k-space line per segment, 94 temporal phases	Extended-phase-graph fitting method	Interleaved monopolar echoes, weakly asymmetric sampling, fatty riot fitting

For a subgroup of acquisitions (belonging to the first part of the study), 56 dynamic single side datasets (ssd) were compared to the average fat fraction^25,26^ and T2 relaxation values^27,28^ of the entire single muscles of the quadriceps (vastus lateralis (VL), vastus intermedius (VI)) from the biomarker study (see Table 1). Single-muscle regions of interest (ROIs) were segmented on the quadriceps by an experienced radiologist.

### Post-processing/statistics

The velocity images were elaborated in Matlab (R2019b [The Mathworks, Inc.]) as in^18,29,30^. The principal strain maps were computed from the displacement maps^17,18^. It was assumed that the pixels of the acquired slice do not move out of the slice during the acquisition.

The positive eigenvalue was considered as the pixel-wise principal strain. For strain calculation, a ROI was drawn on the magnitude image of the PC acquisition including both VL and VI. This broader ROI was chosen to account for the variability in muscle size (i.e., in individuals with thinner muscles, the stimulation effect is expected to be seen not only in the VL but also in deeper muscles such as VI).

As a characteristic value of the eigenvalues of strain, the spatial average over the ROI was calculated for every time frame of the reconstructed single contraction period and the absolute value maximum of this time curve was considered as peak strain. In addition to the maximum principal strain, the difference in respect to the first scan of each subject was calculated as well as the respective difference in the applied current.The slope of linear regression between changes in strain and respective changes in stimulation current was used for normalization.

Finally, the time constants at which the strain reached the maximum (*build-up constant*) and relaxed to zero (*release constant*) were also calculated by fitting the corresponding portions of the strain curve^19^ to a sigmoid curve:

1 $$y = b+\frac{(a-b)}{1+e^{\frac{x-x_0}{\Delta x}}}$$

2 $${\text{Rate}}: \, \frac{\left( a-b \right)}{\Delta x}$$

The rates were firstly calculated as maps and then the mean values of the respective ROIs were estimated.

All graphical plots and statistical analysis were performed with R/RStudio^31^. To correct for dependence of datasets that belonged to the same subject, the repeated measures correlation coefficients were used and Bonferroni correction was applied to the significance levels. Boxplots were partially replaced by scatter plots, if less than 10 data points were available.

The groups of patients and HCs were tested for differences at baseline: in age, body-mass-index (BMI), weight and height. Although with motor point identification the efficiency of the stimulation is standardized and the position of electrodes was registered for follow-ups, it is not possible to exclude minor variations in the repositioning that could be operator dependent or due to differences during motor point identification. Therefore, we also tested for differences in the distance between the electrodes.

The values were ln-transformed to compensate partly for skewness and a two-sided t-test was performed acknowledging not equal variance and not paired data. These comparisons were performed for all datasets at baseline, at time point t1, and for the datasets for which an equal number of data were available at baseline and at time point t1.

Finally, the analysis was exploratory, so no distribution test of the dynamic results was performed.

## Results

The range of the patients’ CSS at baseline was 1 to 4 (19 datasets with (0, 2.5] CSS, [18 datasets (2.5, 3] CSS], [14 datasets (3, 3.5] CSS], [14 datasets (3.5, end] CSS]). The length of the EcoRI fragment was 25.0 ± 5.9 kb. The average values of the dynamometric evaluation were 28.6 ± 13.32 N and of the 6MWT 443.1 ± 138.3 m. There was no indication of distribution differences between HCs and patients in BMI, weight and height (see Supplementary Table S1). However, there were some differences in age at the first follow-up (t1) (p-values < 0.003 = Bonferroni corrected significance level, absolute effect size > 0.8).

Dynamic data could not always be acquired due to patient dropout or technical reasons regarding the stimulation equipment (see Supplementary Table S2 for full data overview). The applied current for NMES had to be often modified for subsequent visits, because of discomfort reported by the subject (see Supplementary Fig. 1). The effective slope of the changes (in reference to the first scan) in current versus changes in strain was 0.00373 (intercept = −0.0069, p = 0.0003). Therefore, a multiplication with 0.00373 was used for normalization to compensate for possible strain dependence on current.

**Figure 1:**
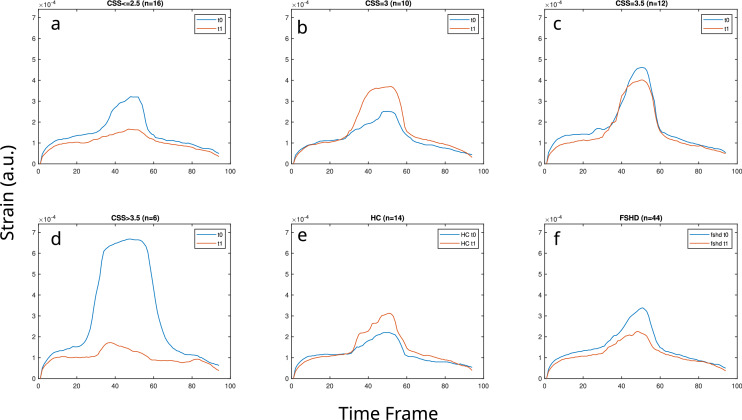
Summary median curves of single side datasets for FSHD patients and healthy controls (HC) for datasets that included acquisitions for both baseline (t0) and time point t1. The patient curves (**a–d,f**) were separately presented for different clinical severity scores (CSS) (**a–d),** as well as cumulatively (**f**). The number of datasets used for every plot is given (n).

**Figure 2:**
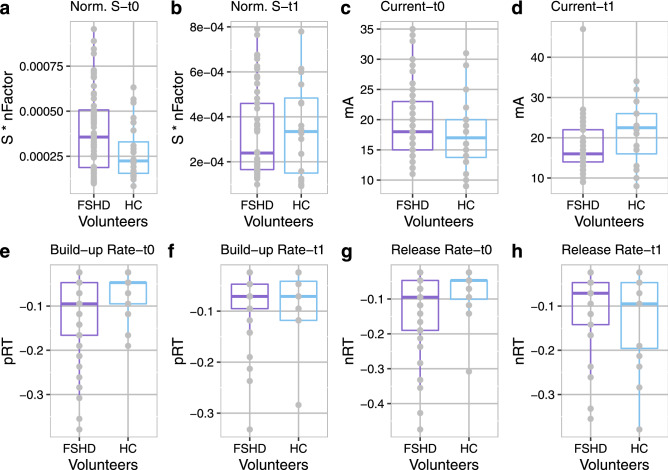
**(a**) Normalized strain (S) at baseline (t0) for FSHD patients and healthy controls (HCs) (FSHD/HCs (1st quantile-Q1) = 1.9e−4/1.6e−4, (median-M) = 3.6e−4/2.2e−4, (3rd quantile-Q3) = 5.1e−4/3.3e−4), (**b**) normalized strain (S) at time point t1 for FSHD patients and HCs ((Q1, M, Q3) for FSHD/HC (1.7e−4/1.5e−4, 2.4e−4/3.3e−4, 4.6e−4/4.8e−4)), (**c**) current applied in mA for t0 (FHSD/HC (Q1, M, Q3) = (15/13.8, 18/17, 23/20) mA, (**d**) current applied in mA for t1 FSHD/HC ((Q1, M, Q3) = (14/16, 16/22.5, 22/26)), (**e**) build-up rates (pRT) at t0: FSHD/HC (Q1, M, Q3) = (−0.17/−0.1, −0.1/−0.05, −0.05/−0.05)), and (**f**) at t1 (−0.1/−0.12, −0.07/−0.07, −0.05/−0.04), (**g**) release-rates (nRT) at t0: nRT-FSHD/HC (−0.19/−0.1, −0.1/−0.05, −0.05/−0.05) and t1 (**h**): nRT-FSHD/HC −0.14/−0.2, −0.07/−0.1, −0.05/−0.05). Here, data is reported as a total with no differentiation. nFactor is the normalization factor for strain.

An overview of the dynamic results in t0 and t1 is presented in Fig. 1 (summary of strain versus time curves) and Fig. 2 (point values summary). Overall, the summary curves of FSHD patients were lower at t1 with the exception of patients with CSS equal to 3 at baseline. For HCs the strain curves had higher values at t1 in comparison to baseline, but similar build-up and release slopes (see Fig. 1). By summarizing the point values (see Fig. 2) in t0, the median, the Q1 and the Q3 of the strain distribution for the FSHD patients were higher than for HCs, as were the Q1 and Q3 of the distribution of the applied current (Fig. 2a,c). Similarly, the values of the median and Q1 for both the build-up and release rates were more negative for the FSHD patients than for the HCs at t0 (Fig. 2e,g). At t1 (Fig. 2b,d), the median and Q3 of the strain of the patients and the applied current were lower than for the HCs. Finally, at t1 the values of the median, Q1 of the release rates for the FSHD patients were less negative in comparison to the HCs, while the median was equal for the build-up rates (Fig. 2f,h).

**Figure 3:**
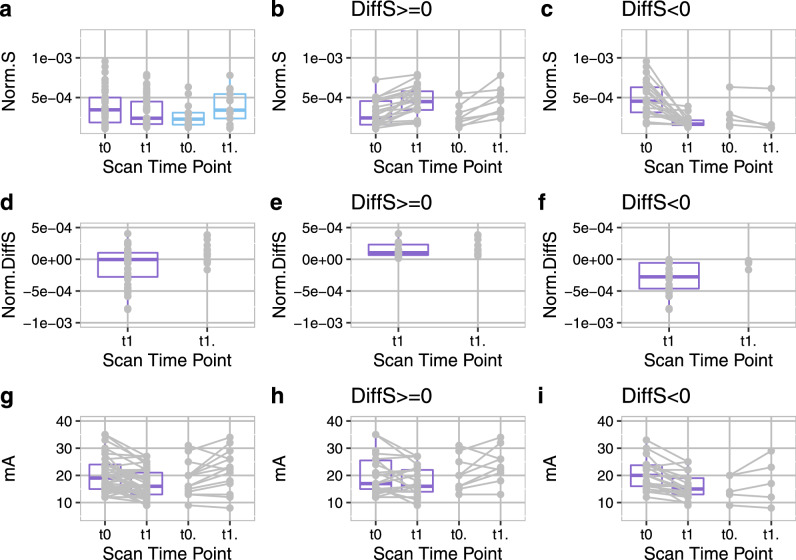
Comparison of FSHD patients (FSHD) and healthy controls (HC): only subjects with equal number of datasets for baseline (t0) and the time point t1 are shown: (**a**) normalized strain in all datasets together (1st column), (**b**) normalized strain in datasets that showed increase of strain at t1 (22 for FSHD, 9 for HCs, 2nd column), (**c)** normalized strain in datasets that showed decrease of strain at t1 (22 for FSHD, 5 for HCs, 3rd column), (**d–f**) Normalized differences of strain (DiffS = Norm.Strain(t1) − Norm.Strain(t0)), for the respective datasets in A–C, **(g–i)** current values (in mA) that were applied in the respective cases. The datasets in the 2nd column (FSHD-Δs+) are very similar to the HCs, while in the 3rd column (FSHD-Δs−) the pronounced decrease in strain differs from HCs.

To minimize data unbalance bias, the comparison between patients and HCs was additionally performed (see Fig. 3) for the groups with the equal number of data points at t0 and t1: 44/14 datasets for the FSHD patients/HC, respectively. For FSHD patients the strain decreased at t1, while for HCs it increased (Fig. 3a). The range of absolute strain values did not differ between the two groups*.* However, the median normalized difference over all datasets of the patients was close to 0 (see Fig. 3a,d). Therefore, for clearer visualization, the results were differentiated based on an increase (Fig. 3b,e,h) or decrease (Fig. 3c,f,i) in strain at the first follow-up (t1). Most of the healthy controls showed a slight increase in strain at t1 (Fig. 3b,e,c,f). Regarding the datasets from patients, half of them had a slight strain increase similar to HCs (Fig. 3b) and half a much more pronounced decrease than the HCs (Fig. 3c). Moreover, the differences in strain between the patients and the HCs seemed to be independent from the applied current (Fig. 3h,i). For the rest of the manuscript, the first patient group (DiffS(t1-t0) >  = 0) will be referred to as FSHD-Δs+ group (i.e., patients’ data with increased strain at t1), while the second (DiffS(t1 − t0) < 0) as FSHD-Δs− ((i.e., patients’ data with decreased strain at t1)).

**Figure 4:**
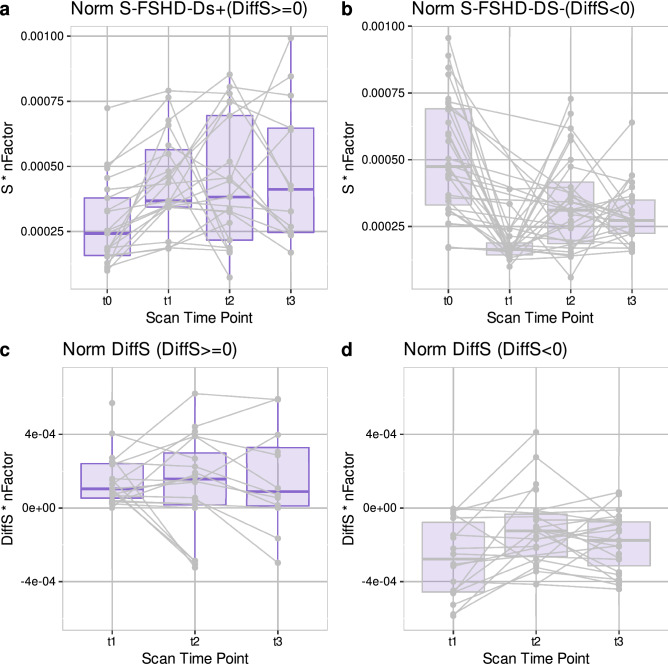
Datasets for FSHD patients, including a minimum of three time points (any combination out of the 4 time points t0, t1, t2, t3). (**a**) Strain for the FSHD-Δs+ datasets (number of datasets:21 (t0), 19 (t1), 21 (t2), 13 (t3))). (**b**) Strain for the FSHD-Δs− datasets (31(t0), 20 (t1), 28 (t2), 25 (t3)) (see Fig. 3). (**c,d**) Respective differences in the strain from t0 (DiffS). For the FSHD-Δs+ group, the median difference from the baseline remains stable (with an increasing variability). For the FSHD-Δs− group, there are larger fluctuations in the difference from the baseline and the absolute difference is larger between the first 2 time points than in subsequent ones. FSHD-Δs+ /FSHD-Δs**−** : groups of the strain data of FSHD patients based on the changes between t0 and t1. Norm.: Values of strain normalized to the applied current.

The datasets of FSHD patients for all time points (t0−t3) were also visualized separately from the HCs since more data points were available for this group (see Fig. 4). To achieve a more representative and unbiased visualization, only datasets for which at least three time points were available were included (e.g. datasets for which only t0 was available were excluded, see Supplementary Table S2 for full data overview). The normalized strain for these datasets and the differences in strain with respect to t0 were visualized after splitting the FSHD-Δs+ /FSHD-Δs− groups (Fig. 4). For the FSHD-Δs+ group there was no clear trend, whereas for the FSHD-Δs− group there was a decreasing trend which was most pronounced between t0 and t1. The median of all datasets between t0 and t3 was decreased by approximately 57%.

**Figure 5:**
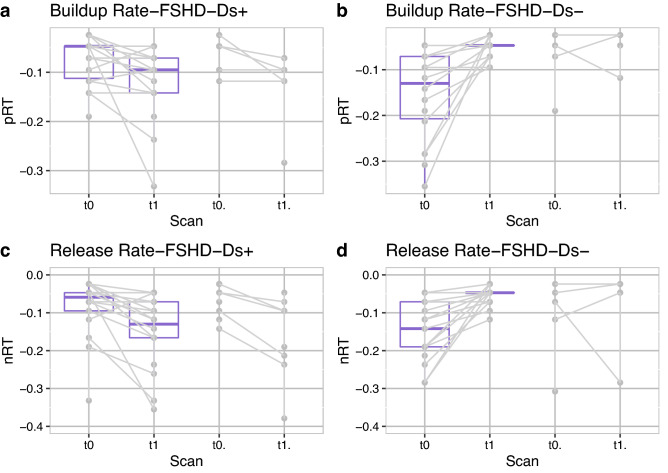
Build-up & release rates comparison between patients and healthy controls (HCs). (**a**) Build-up rate for the first two scans for the FSHD-Δs+ patient group and the HC group (22 for FSHD, 9 for HCs), (**b**) build-up rate for the FSHD-Δs− patient group and the HC group (23 for FSHD, 4 for HCs), (**c**) release rate for the first two scans for the FSHD-Δs+ patient group and the HC group (22 for FSHD, 9 for HCs), (**d**) release rate for the FSHD-Δs− patient group and the HC group (23 for FSHD, 4 for HCs). FSHD-Δs+ /FSHD-Δs− : groups of the strain data of FSHD patients based on the changes between t0 and t1.

The build-up and the release rates, similarly to the strain in the datasets where both t0 and t1 were available, were also analyzed for the splitted groups based on change in the first follow-up (t1). Larger absolute values indicate steeper curves. For the FSHD-Δs+ group (Fig. 5a,c) the rates became more negative in t1 comparably to the HCs (t1.). For the FSHD-Δs− group (Fig. 5b,d) the rates became less negative in t1, a trend that was not observed in the HCs.

**Figure 6:**
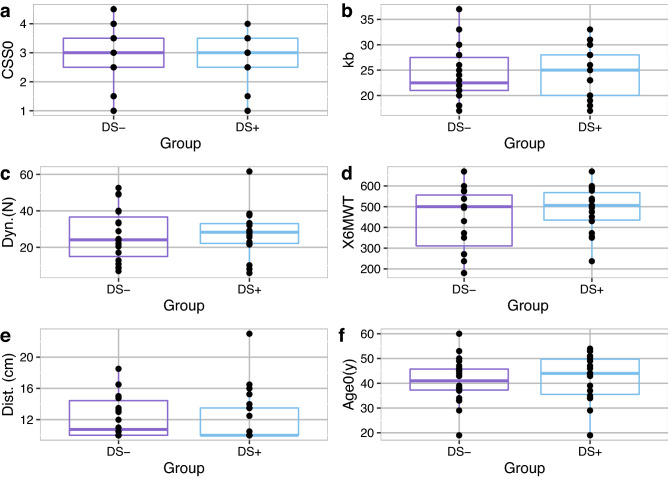
Functional results for the two patient groups FSHD-Δs+ /FSHD-Δs−: **(a)** Clinical Severity score at baseline (CSS0), **(b)** length of the 4q35 BlnI resistant, p13-E11 EcoRI fragment (kb), **(c)** dynamometric evaluation (N), **(d)** 6 min walking test. In addition, the distance between the 2 electrodes **(e)** and the age at baseline **(f)** are given for the two groups. FSHD-Δs+/FSHD-Δs− : groups of the strain data of FSHD patients based on the changes between t0 and t1.

None of the calculated correlation coefficients of strain with CSS, average length of the EcoRI fragment, 6MWT and dynamometry results were strong or significant (correlation coefficient r (p-value): Dynamometry Quadriceps (r = −0.085, p = 0.672), length of the EcoRI fragment (r = −0.27, p = 0.149), CSS (r = −0.199, p = 0.276), 6MWT (r = 0.242, 0.234)). The CSS, average length of the EcoRI fragment, 6MWT and dynamometry results were plotted for the two groups (FSHD-Δs+/FSHD-Δs−, see Fig. 6). The FSHD-Δs+ group had higher median dynamometry measurements and larger EcoRI fragment sizes, whereas there was no difference in the median of the two groups in CSS values and 6MWT results. No differences were observed between the two groups regarding the age at baseline and distance of electrodes. Finally, water T2 and fat fractions values from all available time points were analyzed (i.e., grouped into patient results versus HCs, see Table 2). In addition, the correlation coefficients of strain, build-up, and release rates versus the fat fractions and water T2 values were calculated for all available volunteer datasets as a total (Table 2). There was no significant correlation of T2 or FF values, neither with maximum strain values (p > 0.17), nor with the rates (p > 0.083).

**Table 2:** Correlation coefficients (r) of the strain (S), build-up (pRT) and release rates (nRT) with water T2 (T2) and fat fraction (FF) in the vastus lateralis (VL) and vastus intermedius (VI).

Versus	Correlation Coefficients r (p-value)			Summary values Mean ± standard deviation	
	Strain	Build-up rate	Release rate	T2 (ms)/FF(%) in FSHD patients	T2 (ms)/FF(%) in healthy controls
T2VL	0.204 (0.207)	−0.262 (0.103)	−0.236 (0.143)	43.13 ± 8.61	39.44 ± 2.83
T2VI	0.215 (0.176)	−0.256 (0.107)	−0.202 (0.206)	44.22 ± 7.89	39.20 ± 1.95
FFVL	0.151 (0.339)	−0.184 (0.243)	−0.127 (0.423)	14.59 ± 18.02	6.39 ± 2.54
FFVI	−0.161 (0.309)	0.097 (0.541)	0.27 (0.083)	15.34 ± 18.94	5.01 ± 2.06

The significance level corrected for multiple comparisons was 0.0042. The number of datasets used were: (FSHD patients: 25 datasets (t0), 17(t1), 9(t2) & healthy controls (7(t0.))).

## Discussion

In this study, we explored the applicability and potential role of dynamic muscle MRI in the field of neuromuscular diseases, applying it to a population of FSHD subjects and HCs over a two-year period. The acquisition protocol, with specific regard to the electrical stimulation during the MRI scan, was well tolerated by FSHD subjects. By dividing the results based on strain increase/ decrease at the first follow-up (Δs + /Δs−), the FSHD-Δs- group not only showed decreasing strain (in both magnitude and rates) but also slightly lower dynamometric results and smaller fragment size. However, the FSHD-Δs− group had similar CSS values and 6MWT results with the FSHD-Δs+ group. Further conclusions cannot be made due to the limited data and the exploratory type of the analysis.

T2 and FF were not correlated to the absolute maximum strain value or rates suggesting that the dynamic results could provide complementary information on skeletal muscle tissue composition, and potentially on fibrosis. Yet further experiments are needed to investigate this hypothesis.

Regarding stimulation intensity, for various HCs the applied current was higher at t1 than at t0, presumably due to exaggerated perceived discomfort at t0, which prevented reaching the targeted intensity. In subsequent visits higher currents were actually well tolerated. This habituation effect could contribute to the lower strain of controls compared to the FSHD group at baseline and the higher strain of the control group at t1. In the subgroup analysis, this effect could also be a possible explanation for the slight increase in the strain values observed in the HC and FSHD-Δs+ groups. These cases, many of which correspond to patients with a CSS of 3, could also be expressing a similar habituation pattern to healthy volunteers. Further investigation is needed to establish whether this is the case and why. Finally, no dependence of the build-up and release rates on the applied current was observed.

Most previous similar MRI studies focussed on comparing young and senior healthy individuals^19,32,33^ and highlighting differences in muscle composition. To our knowledge, there is no study applying such a method to a cohort of patients affected by neuromuscular diseases. Here, the results of dynamic MRI of the quadriceps muscle contraction were different in the longitudinal evaluation for FSHD patients with respect to HCs. Surprisingly, the range of neither the strain values nor the build-up and release rates considerably differ between patients and controls^19^. Moreover, in comparison to previous studies^34^, the increase of the necessary current is unexpected, which could be explained by various factors including differences in participants’ age and scan time interval. Indeed, a previous study investigating the reproducibility of MR imaging of NMES-evoked contractions during plantar flexion in healthy volunteers showed a similar increasing trend of the strain when the force output was kept constant^18^.

There were a few limitations in this preliminary study. First, the investigated muscles were chosen based on the robustness and replicability^17,19^ of the measurements. Second, the number of participants was relatively low, not perfectly balanced between HCs and patients and there were missing data in later time points for various reasons. Due to the missing follow-up data, the age match between patients and controls was lost at subsequent time points. However, this was a pilot study and the number of available data was sufficient for various comparisons between patients and control subjects. Third, there was no measurement of the NMES-evoked force to standardize the stimulus, since this would have required additional MR-compatible equipment. Therefore, we designed the protocol to induce a submaximal isometric contraction defined as the “minimum visible muscle twitch”, so that no measurable force output would be expected. This setting was in line with^19^, where differences in strain between senior and younger volunteers were observed in strain with the same protocol. In addition, force measurement in the scanner is particularly complicated in the case of knee extension. To ensure reproducibility over time, the position of the electrodes was standardized by measuring the exact position of the electrodes at every visit and using a simulation pen for motor point localization. When possible, the current of the stimulation remained the same, under the condition however that a contraction was induced and the volunteer was not in discomfort. Nevertheless, the current had to be occasionally modified and a bias due to this cannot be completely excluded when evaluating strain, but the normalized changes in strain were also calculated and were expected to be independent from changes in the current.

It should be noted that the current setup was mainly dictated by the technical possibilities available when the study was initiated; recent advances in the acquisition method now enable three-dimensional coverage of the muscles^18,35^ and might offer further flexibility and diagnostic value, thus warranting further investigation. However, it is a technical setup of low additional cost (to the MRI examination), which requires a relatively short preparation time and is particularly adequate for progress studies (using the subject as their own control).

In summary, our results suggest that evaluating the dynamic phase contrast images of evoked muscle contraction could provide information about the evolution of FSHD at a single muscle level and this could be potentially applied to other myopathies. This applies to mostly superficial muscles and a longitudinal follow-up of a disease. While a cross-sectional comparison is possible, it was not feasible with the existing data and further studies are needed. The quantitative results of strain MRI through evoked NMES could complement a standard quantitative MRI protocol, possibly highlighting subtle changes before they become detectable with other quantitative MRI biomarkers.

## Supplementary Information

Supplementary Information 1.

Dataset S1.

Supplementary Information 2.

Supplementary Legends.

Supplementary Information 3.

Supplementary Information 4.

## Abbreviations

FF: Fat fraction
FSHD: Facioscapulohumeral muscular dystrophy
FSHD-Δs+: Patients that show increased strain in the comparison between healthy controls and FSHD patients for the first two time points
FSHD-Δs−: Patients that show decreased strain in the comparison between healthy controls and FSHD patients for the first two time points
HCs: Healthy controls
NMES: Neuromuscular electrical stimulation
PC: Phase contrast
pRT: Build-up
nRT: Release rates
ROI: Region of interest
S: Strain
ssd: Single side datasets
STIR: Short tau inversion recovery
t0, t1, t2, t3: 1st (baseline), 2nd, 3rd, and 4th study time points for FSHD patients
t0., t1., t2.: 1 (baseline), 2nd, and 3rd study time points for healthy controls
TR/TE: Repetition time/echo time
TENS: Transcutaneous electrical nerve stimulation
VL: Vastus lateralis (muscle)
VI: Vastus intermedius (muscle)
6MWT: 6-Minute walking test
Q1/Q3: 1st and 3rd quantile
