# Supplementary material for: Dynamic magnetic resonance imaging of muscle contraction in facioscapulohumeral muscular dystrophy
Source: Sci Rep. 2022 May 4;12:7250. doi: 10.1038/s41598-022-11147-2 (PMC9068910; doi:10.1038/s41598-022-11147-2)
Supplement: Supplementary file 1 — Supplementary Information 1. [file 41598_2022_11147_MOESM1_ESM.pdf]

TITLE: Dynamic magnetic resonance imaging of muscle contraction in facioscapulohumeral muscular dystrophy

Xeni Deligianni[1,2], Francesco Santini[1,2*], Matteo Paoletti[3], Francesca Solazzo[4], Niels Bergsland[5,6], Giovanni Savini[3], Arianna Faggioli[3], Giancarlo Germani[3], Mauro Monforte[7], Enzo Ricci[7], Giorgio Tasca[7§] and Anna Pichiecchio[3,8§]

Supplementary Material Submission ID 464d1b5c-3332-4d24-8b5f-1727b87e9c59

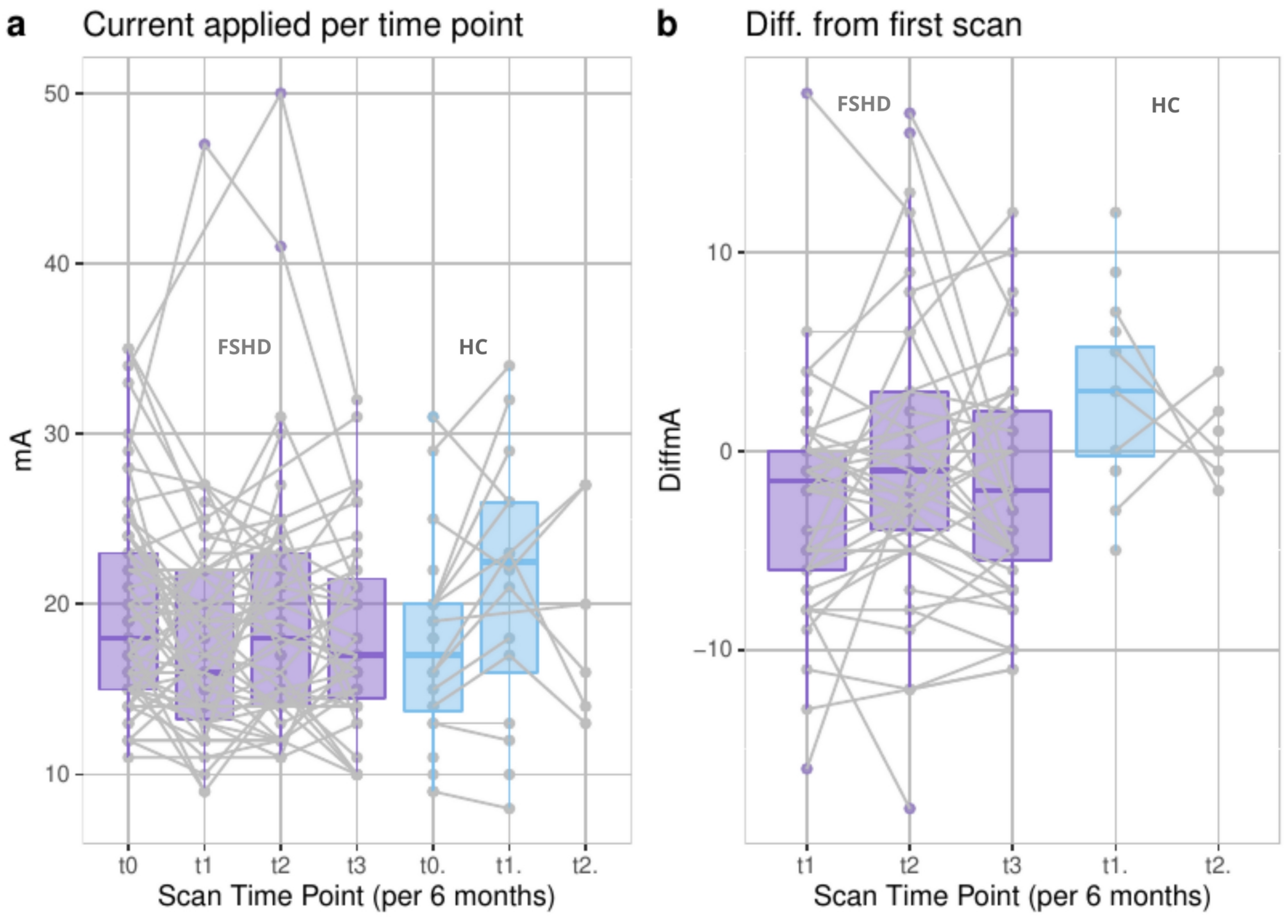

Supplementary Figure 1. Visualization of the settings of current for every respective scan in absolute values as well as differences (from the first scan). **a**) Current per time point (*left*: t0-t3 for FSHD patients, *right*: t0.-t2. for healthy controls), **b**) Differences in current in mA from t0 calculated for every subject individually. The current was generally slightly decreased from t0, with the median current changes of -1.5 mA (*t1*), -1 mA (*t2*), and - 2mA (*t3*). For HCs the median increase of current between the first and the second scan was + 3mA (*t1.*)

FSHD/ HCs	Age at baseline(t0)	BMI	Weight	Height	Distance of electrodes
Baseline (t0) all datasets	0.2086 (-0.34) d=-4 y	0.4641 (0.165) d=2.7 BMI	0.0745 (0.343) d=7 kg	0.0329 (0.482) d=0 cm	0.0068 (0.661) d=2 cm
t1(all datasets)	0.0003 (-0.926) d=-7 y	0.293 (-0.302) d=0.45BMI	0.8783 (-0.037) d=0 kg	0.0656 (0.508) d=3 cm	0.4737 (-0.238) d=0 cm
Datasets with both t0&t1	0 (-1.167) d=-9 y	0.0967 (-0.32) d=-0.95 BMI	0.5054 (-0.105) d=-7 kg	0.0791 (0.348) d=2.5 cm	0.6945 (NA) d=0 cm

Supplementary Table S1. p-values from the t-tests (on log transformed distributions) of the comparison between FSHD patients and healthy controls (Hcs), as well as Cohen's effect size (in brackets) and difference (d) in the medians. The level of significance after Bonferroni correction was 0.003. BMI: Body-Mass-Index.

		Time Points (6-month interval)	**t0**	**t1**	**t2**	**t3**
FSHD Patients		Number of subjects	34	24	26	20
	Single Side Datasets (ssd)	Nr. of ssd (201 in total)	65	46	51	39
		Nr. of ssd with exactly **1** time point	6	0	1	0
		Nr. of ssd with exactly **2** time points	7	7	1	1
		Nr. of ssd with exactly **3** time points	33	20	30	19
		Nr. of ssd with exactly **4** time points	19	19	19	19
		Nr. of ssd with **min 2** time points	59	46	50	39
		Nr. of ssd with **min 3** time points	52	39	49	38
		Equal number of points in t0 and t1	44			
Healthy Controls		Number of subjects	13	10	3	
	Single Side Datasets (ssd)	Nr. of ssd (46 in total)	24	16	6	
		Nr. of ssd with exactly **1** time point	9	1	0	
		Nr. of ssd with exactly **2** time points	11	11	2	
		Nr. of ssd with exactly **3** time points	4	4	4	
		Nr. of ssd with **min 2** time points	15	15	6	
		Equal number of points in t0 and t1	14			

Supplementary Table S2. Overview of the number of dynamic datasets included in the analysis at the various time points: baseline (t0), 6 months interval (t1), 12 months interval (t2), 24 months interval (t3).
