# Supplementary material for: Dynamic magnetic resonance imaging of muscle contraction in facioscapulohumeral muscular dystrophy
Source: Sci Rep. 2022 May 4;12:7250. doi: 10.1038/s41598-022-11147-2 (PMC9068910; doi:10.1038/s41598-022-11147-2)
Supplement: Supplementary file 3 — Supplementary Information 2. [file 41598_2022_11147_MOESM3_ESM.pdf]

````
---
output:
  pdf_document: default
---

<!-- Kommentare sind moeglich mit HTML Kommentaren
<!-- Based on knitr-minimal.Rmd on http://yihui.name/knitr/demo/minimal/ --
>

# Figures for FSHD Dynamic Analysis Manuscripts

The data used are produced with FSHD_Rscript.R and saved as
workspaceFSHD_vs1.RData.

## R Code

### Code Chunks

We can set the global options as follows:

```{r setup}
## set global chunk options: all images will be 7x5 inches
knitr::opts_chunk$set(fig.width = 7, fig.height = 5)
options(digits = 4)
```

Change baseDir to your directory.

```{r plot1, message=FALSE, warning=FALSE, echo=FALSE}
baseDir='/myDir/';

#my_dat = paste(baseDir, "dataFSHD.csv", sep="" )
load("workspaceFSHD_vs1.RData")

library(ggplot2)
library(ggpubr)
c2="skyblue2"
col1="mediumpurple3"
levels(dat$Scan) <- c(levels(dat$Scan), "t0.")
levels(dat$Scan) <- c(levels(dat$Scan), "t1.")
levels(dat$Scan) <- c(levels(dat$Scan), "t2.")
dat[dat$Scan == "T0",]$Scan <- "t0."
dat[dat$Scan == "T1",]$Scan <- "t1."
dat[dat$Scan == "T2",]$Scan <- "t2."

#Supplementary Figure 1-Current
a1<-ggplot(data = dat, mapping = aes(y = mA, x = Scan,col="gray")) +#
````
````

~~~
  geom_point(col="gray")
+geom_boxplot(alpha=c(0.5,0.5,0.5,0.5,0.5,0.5,0),col=c(col1,col1,col1,col1,c2,c2,NA),fil
+geom_line(aes(group=IDL),col="gray")+xlab("Scan Time Point (per 6 months)")+

  ggtitle("Current applied per time point")+
  theme(panel.background = element_rect(fill = 'white',colour='gray'))+
  theme(panel.grid.major = element_line(colour = "gray"))

b1<-ggplot(data = dat, mapping = aes(y = DiffmA, x = Scan,col="gray")) +
   geom_point(col="gray")
+geom_boxplot(alpha=c(0.5,0.5,0.5,0.5,0),col=c(col1,col1,col1,c2,NA),fill=c(col1,col1,co
+
   geom_line(aes(group=IDL),col="gray")+xlab("Scan Time Point (per 6
months)")+
   scale_x_discrete(limits = c("t1","t2","t3","t1.","t2."))+
  ggtitle("Diff. from first scan")+
    theme(panel.background = element_rect(fill = 'white',colour='gray'))
+theme(panel.grid.major = element_line(colour = "gray"))

g1<-ggarrange(a1, b1, labels = c("a","b"),
                    ncol = 2, nrow = 1)
ggsave(g1, file="figure1.eps", device="eps")
```

```{r plot2, message=FALSE, warning=FALSE, echo=FALSE}
baseDir='/myDir/';

load("workspaceFSHD_vs1.RData")

library(ggplot2)
library(ggpubr)
#Figure 2-Volunteers versus patients
levels(dat$PV) <- c(levels(dat$PV), "FSHD")
levels(dat$PV) <- c(levels(dat$PV), "HC")
dat[dat$PV == "0",]$PV <- "FSHD"
dat[dat$PV == "1",]$PV <- "HC"

col2="skyblue2"
col1="mediumpurple3"
a2<-ggplot (aes(x= PV, y=S*nFactor),data=dat[dat$TP==0,])
+xlab("Volunteers")+
   geom_boxplot(alpha=c(0.2,0.2),col=c(col1,col2),fill=c(col1,col2))+
   geom_point(col="gray")+
   ggtitle("Norm. S-t0")+theme(plot.title = element_text(size = 10))
+#ylim(min(s1[1],s2[1],s3[1],s4[1]),max(s1[5],s2[5],s3[5],s4[5]))+
  theme(panel.background = element_rect(fill = 'white',colour='gray'))
+theme(panel.grid.major = element_line(colour = "gray"))

  #geom_hline(yintercept=6.28e-05)
~~~

```
b2<-ggplot (aes(x= PV, y=S*nFactor),data=dat[dat$TP==1,])
+xlab("Volunteers")+
   geom_boxplot(alpha=c(0.2,0.2),col=c(col1,col2),fill=c(col1,col2))+
   geom_point(col="gray")
+#ylim(min(s1[1],s2[1],s3[1],s4[1]),max(s1[5],s2[5],s3[5],s4[5]))+
   ggtitle("Norm. S-t1")+theme(plot.title = element_text(size = 10))+
  theme(panel.background = element_rect(fill = 'white',colour='gray'))
+theme(panel.grid.major = element_line(colour = "gray"))

c2<-ggplot (aes(x= PV, y=mA),data= dat[dat$TP==0,])+xlab("Volunteers")+
   geom_boxplot(alpha=c(0.2,0.2),col=c(col1,col2),fill=c(col1,col2))
+#ylim(min(s5[1],s6[1],s7[1],s8[1]),max(s5[5],s6[5],s7[5],s8[5]))+
   geom_point(col="gray")+
   ggtitle("Current-t0")+theme(plot.title = element_text(size = 10))+
  theme(panel.background = element_rect(fill = 'white',colour='gray'))
+theme(panel.grid.major = element_line(colour = "gray"))

d2<-ggplot (aes(x= PV, y=mA),data= dat[dat$TP==1,])+xlab("Volunteers")+
   geom_boxplot(alpha=c(0.2,0.2),col=c(col1,col2),fill=c(col1,col2))
+#ylim(min(s5[1],s6[1],s7[1],s8[1]),max(s5[5],s6[5],s7[5],s8[5]))+
   geom_point(col="gray")+
   ggtitle("Current-t1")+theme(plot.title = element_text(size = 10))+
  theme(panel.background = element_rect(fill = 'white',colour='gray'))
+theme(panel.grid.major = element_line(colour = "gray"))

e2<-ggplot (aes(x= PV, y=pRT),data=dat[dat$TP==0,])+xlab("Volunteers")+
   geom_boxplot(alpha=c(0.2,0.2),col=c(col1,col2),fill=c(col1,col2))
+#ylim(min(s9[1],s10[1],s11[1],s12[1]),max(s9[5],s10[5],s11[5],s12[5]))+
   geom_point(col="gray")+
   ggtitle("Build-up Rate-t0")+theme(plot.title = element_text(size = 10))+
  theme(panel.background = element_rect(fill = 'white',colour='gray'))
+theme(panel.grid.major = element_line(colour = "gray"))

f2<-ggplot (aes(x= PV, y=pRT),data=dat[dat$TP==1,])+xlab("Volunteers")+
   geom_boxplot(alpha=c(0.2,0.2),col=c(col1,col2),fill=c(col1,col2))
+#ylim(min(s9[1],s10[1],s11[1],s12[1]),max(s9[5],s10[5],s11[5],s12[5]))+
   geom_point(col="gray")+
   ggtitle("Build-up Rate-t1")+theme(plot.title = element_text(size = 10))+
  theme(panel.background = element_rect(fill = 'white',colour='gray'))
+theme(panel.grid.major = element_line(colour = "gray"))

g2<-ggplot (aes(x=PV, y=nRT),data=dat[dat$TP==0,])+xlab("Volunteers")+
   geom_boxplot(alpha=c(0.2,0.2),col=c(col1,col2),fill=c(col1,col2))
+#ylim(min(s13[1],s14[1],s15[1],s16[1]),max(s13[5],s14[5],s15[5],s16[5]))+
   geom_point(col="gray")+
   ggtitle("Release Rate-t0")+theme(plot.title = element_text(size = 10))+
  theme(panel.background = element_rect(fill = 'white',colour='gray'))
+theme(panel.grid.major = element_line(colour = "gray"))
```

````
h2<-ggplot (aes(x=PV, y=nRT),data=dat[dat$TP==1,])+xlab("Volunteers")+
   geom_boxplot(alpha=c(0.2,0.2),col=c(col1,col2),fill=c(col1,col2))
+#ylim(min(s13[1],s14[1],s15[1],s16[1]),max(s13[5],s14[5],s15[5],s16[5]))+
   geom_point(col="gray")+
   ggtitle("Release Rate-t1")+theme(plot.title = element_text(size = 10))+
  theme(panel.background = element_rect(fill = 'white',colour='gray'))
+theme(panel.grid.major = element_line(colour = "gray"))


fig2<-ggarrange(a2, b2, c2, d2,e2,f2,g2,h2,labels =
c("a","b","c","d","e","f","g","h"),
          ncol =4, nrow = 2)
ggsave(fig2, file="figure2.eps", device="eps")

```



```{r plot3, message=FALSE, warning=FALSE, echo=FALSE}
baseDir='/myDir/';

#my_dat = paste(baseDir, "dataFSHD.csv", sep="" )
load("workspaceFSHD_vs1.RData")

library(ggplot2)
library(ggpubr)
library(gridExtra)
#Figure 3-Volunteers versus patients
c2="skyblue2"
col1="mediumpurple3"

levels(datPV$Scan) <- c(levels(datPV$Scan), "t0.")
levels(datPV$Scan) <- c(levels(datPV$Scan), "t1.")

datPV[datPV$Scan == "T0",]$Scan <- "t0."
datPV[datPV$Scan == "T1",]$Scan <- "t1."


levels(datPV_P$Scan) <- c(levels(datPV_P$Scan), "t0.")
levels(datPV_P$Scan) <- c(levels(datPV_P$Scan), "t1.")

datPV_P[datPV_P$Scan == "T0",]$Scan <- "t0."
datPV_P[datPV_P$Scan == "T1",]$Scan <- "t1."


levels(datPV_N$Scan) <- c(levels(datPV_N$Scan), "t0.")
levels(datPV_N$Scan) <- c(levels(datPV_N$Scan), "t1.")

datPV_N[datPV_N$Scan == "T0",]$Scan <- "t0."
````

```
datPV_N[datPV_N$Scan == "T1",]$Scan <- "t1."


a3<-ggplot (aes(x= Scan, y=S*nFactor),data=datPV)+xlab("Scan Time Point")
+ylab("Norm.S")+
   geom_boxplot(alpha=c(0.2,0.2,0.2,0.2),col=c(col1,col1,c2,c2),fill=c(col1,col1,c2,c2))
+ylim(0.0001,0.0013)+
   geom_point(col="gray")+geom_line(aes(group=IDL),col="gray",alpha=0.15)+
  theme(panel.background = element_rect(fill = 'white',colour='gray'))
+theme(panel.grid.major = element_line(colour = "gray"))

b3<-ggplot (aes(x= Scan, y=S*nFactor),data=datPV_P)+xlab("Scan Time Point")
+ylab("Norm.S")+
   geom_boxplot(alpha=c(0.2,0.2,0,0),col=c(col1,col1,NA,NA),fill=c(col1,col1,c2,c2))
+ylim(0.0001,0.0013)+
   geom_point(col="gray")+geom_line(aes(group=IDL),col="gray")+
   ggtitle("DiffS>=0")+
  theme(panel.background = element_rect(fill = 'white',colour='gray'))
+theme(panel.grid.major = element_line(colour = "gray"))

c3<-ggplot (aes(x= Scan, y=S*nFactor),data=datPV_N)+xlab("Scan Time Point")
+ylim(0.0001,0.0013)+ylab("Norm.S")+
   geom_boxplot(alpha=c(0.2,0.2,0,0),col=c(col1,col1,NA,NA),fill=c(col1,col1,c2,c2))
+
   geom_point(col="gray")+geom_line(aes(group=IDL),col="gray")+
   ggtitle("DiffS<0")+
  theme(panel.background = element_rect(fill = 'white',colour='gray'))
+theme(panel.grid.major = element_line(colour = "gray"))

d3<-ggplot (aes(x= Scan, y=DiffS*nFactor),data=datPV[datPV$TP>0,])
+xlab("Scan Time Point")+ylab("Norm.DiffS")+
  geom_boxplot(alpha=c(0.2,0),col=c(col1,NA),fill=c(col1,c2))+
scale_x_discrete(limits = c("t1","t1."))+
  geom_point(col="gray")+ylim(-0.001,0.0005)+
  theme(panel.background = element_rect(fill = 'white',colour='gray'))
+theme(panel.grid.major = element_line(colour = "gray"))

e3<-ggplot (aes(x= Scan, y=DiffS*nFactor),data=datPV_P[datPV_P$TP>0,])
+xlab("Scan Time Point")+ylab("Norm.DiffS")+
  geom_boxplot(alpha=c(0.2,0),col=c(col1,NA),fill=c(col1,c2))+
scale_x_discrete(limits = c("t1","t1."))+
  geom_point(col="gray")+ylim(-0.001,0.0005)+
  ggtitle("DiffS>=0")+
  theme(panel.background = element_rect(fill = 'white',colour='gray'))
+theme(panel.grid.major = element_line(colour = "gray"))

f3<-ggplot (aes(x= Scan, y=DiffS*nFactor),data=datPV_N[datPV_N$TP>0,])
+xlab("Scan Time Point")+ylab("Norm.DiffS")+
  geom_boxplot(alpha=c(0.2,0),col=c(col1,NA),fill=c(col1,c2))+
```

```
  geom_point(col="gray")+ylim(-0.001,0.0005)+
  ggtitle("DiffS<0")+
  theme(panel.background = element_rect(fill = 'white',colour='gray'))
+theme(panel.grid.major = element_line(colour = "gray"))

g3<-ggplot (aes(x= Scan, y=mA),data=datPV)+xlab("Scan Time Point")+
   geom_boxplot(alpha=c(0.2,0.2,0,0),col=c(col1,col1,NA,NA),fill=c(col1,col1,c2,c2))
+
   geom_point(col="gray")+geom_line(aes(group=IDL),col="gray")+ylim(5,40)+
  theme(panel.background = element_rect(fill = 'white',colour='gray'))
+theme(panel.grid.major = element_line(colour = "gray"))

h3<-ggplot (aes(x= Scan, y=mA),data=datPV_P)+xlab("Scan Time Point")+
   geom_boxplot(alpha=c(0.2,0.2,0,0),col=c(col1,col1,NA,NA),fill=c(col1,col1,c2,c2))
+
   geom_point(col="gray")+geom_line(aes(group=IDL),col="gray")+ylim(5,40)+
   ggtitle("DiffS>=0")+
  theme(panel.background = element_rect(fill = 'white',colour='gray'))
+theme(panel.grid.major = element_line(colour = "gray"))

i3<-ggplot (aes(x= Scan, y=mA),data=datPV_N)+xlab("Scan Time Point")+
   geom_boxplot(alpha=c(0.2,0.2,0,0),col=c(col1,col1,NA,NA),fill=c(col1,col1,c2,c2))
+
   geom_point(col="gray")+geom_line(aes(group=IDL),col="gray")+ylim(5,40)+
   ggtitle("DiffS<0")+
  theme(panel.background = element_rect(fill = 'white',colour='gray'))
+theme(panel.grid.major = element_line(colour = "gray"))


g3<-ggarrange(a3, b3,c3,d3,e3,f3,g3,h3,i3, labels =
c("a","b","c","d","e","f","g","h","i"),align = "hv",
          ncol = 3, nrow = 3)
ggsave(g3, file="figure3.eps", device="eps")

```

```
```{r plot4, message=FALSE, warning=FALSE, echo=FALSE}
baseDir='/myDir/';

#my_dat = paste(baseDir, "dataFSHD.csv", sep="" )
load("workspaceFSHD_vs1.RData")
#install.packages("Cairo")
library(ggplot2)
library(ggpubr)
c2="skyblue2"
col1="mediumpurple3"

levels(dat$Scan) <- c(levels(dat$Scan), "t0.")
levels(dat$Scan) <- c(levels(dat$Scan), "t1.")
```
```

```
levels(dat$Scan) <- c(levels(dat$Scan), "t2.")
dat[dat$Scan == "T0",]$Scan <- "t0."
dat[dat$Scan == "T1",]$Scan <- "t1."
dat[dat$Scan == "T2",]$Scan <- "t2."

#Figure 4-patients only
a4<-ggplot (aes(x= Scan,
y=S*nFactor),data=dat_sub_min3p[dat_sub_min3p$PV=="0"&dat_sub_min3p$Group=="P",])
+xlab("Scan Time Point")+
  geom_boxplot(alpha=c(0.2,0.2,0.2,0.2),col=c(col1,col1,col1,col1),fill=c(col1,col1,col1
+
  geom_point(col="grey")+
  geom_line(aes(group=IDL),col="grey",alpha=0.9)+
  ggtitle("Norm S-FSHD-Ds+(DiffS>=0)")+
  theme(panel.background = element_rect(fill = 'white',colour='gray'))
+theme(panel.grid.major = element_line(colour = "gray"))

b4<-ggplot (aes(x= Scan,
y=S*nFactor),data=dat_sub_min3p[dat_sub_min3p$PV=="0"&dat_sub_min3p$Group=="N",])
+xlab("Scan Time Point")+
  geom_boxplot(alpha=0.2,col="grey",fill=col1)+
  geom_point(col="grey")+
  geom_line(aes(group=IDL),col="grey",alpha=0.9)+
  ggtitle("Norm S-FSHD-DS-(DiffS<0)")+
  theme(panel.background = element_rect(fill = 'white',colour='gray'))
+theme(panel.grid.major = element_line(colour = "gray"))

c4<-ggplot (aes(x= Scan,
y=DiffS*nFactor),data=dat_sub_min3p[dat_sub_min3p$PV=="0"&dat_sub_min3p$Group=="P",])
+xlab("Scan Time Point")+
  geom_boxplot(alpha=c(0.2,0.2,0.2),col=c(col1,col1,col1),fill=c(col1,col1,col1))
+ scale_x_discrete(limits = c("t1","t2","t3"))+
  geom_point(col="grey")+ylim(-0.0006,0.0007)+
 geom_line(aes(group=IDL),col="grey",alpha=0.9)+
  ggtitle("Norm DiffS (DiffS>=0)")+
  theme(panel.background = element_rect(fill = 'white',colour='gray'))
+theme(panel.grid.major = element_line(colour = "gray"))

d4<-ggplot (aes(x= Scan,
y=DiffS*nFactor),data=dat_sub_min3p[dat_sub_min3p$PV=="0"&dat_sub_min3p$Group=="N",])
+xlab("Scan Time Point")+
  geom_boxplot(alpha=0.2,col="grey",fill=col1)+ scale_x_discrete(limits =
c("t1","t2","t3"))+
  geom_point(col="grey")+ylim(-0.0006,0.0007)+
 geom_line(aes(group=IDL),col="grey",alpha=0.9)+
  ggtitle("Norm DiffS (DiffS<0)")+
  theme(panel.background = element_rect(fill = 'white',colour='gray'))
+theme(panel.grid.major = element_line(colour = "gray"))
```

````
g4<-ggarrange(a4, b4, c4, d4,labels = c("a","b","c","d"),
          ncol = 2, nrow = 2)
ggsave(filename="figure4.eps",plot = print(g4),dpi="print" (300),
device=cairo_ps("figure4.eps",fallback_resolution=600))

```
```
```{r plot5, message=FALSE, warning=FALSE, echo=FALSE}
baseDir='/myDir/';

#my_dat = paste(baseDir, "dataFSHD.csv", sep="" )
load("workspaceFSHD_vs1.RData")


library(ggplot2)
library(ggpubr)
c2="skyblue2"
col1="mediumpurple3"

levels(datPV$Scan) <- c(levels(datPV$Scan), "t0.")
levels(datPV$Scan) <- c(levels(datPV$Scan), "t1.")

datPV[datPV$Scan == "T0",]$Scan <- "t0."
datPV[datPV$Scan == "T1",]$Scan <- "t1."


levels(datPV_P$Scan) <- c(levels(datPV_P$Scan), "t0.")
levels(datPV_P$Scan) <- c(levels(datPV_P$Scan), "t1.")

datPV_P[datPV_P$Scan == "T0",]$Scan <- "t0."
datPV_P[datPV_P$Scan == "T1",]$Scan <- "t1."


levels(datPV_N$Scan) <- c(levels(datPV_N$Scan), "t0.")
levels(datPV_N$Scan) <- c(levels(datPV_N$Scan), "t1.")

datPV_N[datPV_N$Scan == "T0",]$Scan <- "t0."
datPV_N[datPV_N$Scan == "T1",]$Scan <- "t1."
#Figure 5 Extra-Negative & Positive rate
a5<-ggplot (aes(x= Scan, y=pRT),data=datPV[datPV$Group=="P",])+
  geom_boxplot(alpha=c(0.2,0.2,0,0),col=c(col1,col1,NA,NA),fill=c(col1,col1,c2,c2))
+#alpha=0.2,col=c(2,2,4,4),fill=c(2,2,4,4)
  geom_point(col="grey")+
  geom_line(aes(group=IDN),col="lightgray")+
  ggtitle("Buildup Rate-FSHD-Ds+")+
  theme(panel.background = element_rect(fill = 'white',colour='gray'))
+theme(panel.grid.major = element_line(colour = "gray"))

b5<-ggplot (aes(x= Scan, y=pRT),data=datPV[datPV$Group=="N",])+
````
````

~~~
  geom_boxplot(alpha=c(0.2,0.2,0,0),col=c(col1,col1,NA,NA),fill=c(col1,col1,c2,c2))
+#alpha=0.2,col=c(2,2,4,4),fill=c(2,2,4,4)
  geom_point(col="grey")+
  geom_line(aes(group=IDN),col="lightgray")+
  ggtitle("Buildup Rate-FSHD-Ds-")+
  theme(panel.background = element_rect(fill = 'white',colour='gray'))
+theme(panel.grid.major = element_line(colour = "gray"))

c5<-ggplot (aes(x= Scan, y=nRT),data=datPV[datPV$Group=="P",])+
  geom_boxplot(alpha=c(0.2,0.2,0,0),col=c(col1,col1,NA,NA),fill=c(col1,col1,c2,c2))
+#alpha=0.2,col=c(2,2,4,4),fill=c(2,2,4,4)
  geom_point(col="grey")+ylim(-0.4,0)+
  geom_line(aes(group=IDN),col="lightgray")+
   ggtitle("Release Rate-FSHD-Ds+")+
   theme(panel.background = element_rect(fill = 'white',colour='gray'))
+theme(panel.grid.major = element_line(colour = "gray"))

d5<-ggplot (aes(x= Scan, y=nRT),data=datPV[datPV$Group=="N",])+
  geom_boxplot(alpha=c(0.2,0.2,0,0),col=c(col1,col1,NA,NA),fill=c(col1,col1,c2,c2))
+#alpha=0.2,col=c(2,2,4,4),fill=c(2,2,4,4)
  geom_point(col="grey")+ylim(-0.4,0)+
  geom_line(aes(group=IDN),col="lightgray")+
   ggtitle("Release Rate-FSHD-Ds-")+
   theme(panel.background = element_rect(fill = 'white',colour='gray'))
+theme(panel.grid.major = element_line(colour = "gray"))

g5<-ggarrange(a5, b5, c5, d5, labels = c("a","b","c","d"),
          ncol = 2, nrow = 2)
ggsave(g5, file="figure5.eps", device="eps")
```

```{r plot6, message=FALSE, warning=FALSE, echo=FALSE}
baseDir='/myDir/'

#my_dat = paste(baseDir, "dataFSHD.csv", sep="" )
load("workspaceFSHD_vs1.RData")
#datPV_posfilt<-datPV[datPV$pDxE<1500,]
#datPV_negfilt<-datPV[datPV$nDxE<1500,]

library(ggplot2)
library(ggpubr)
#Figure 6-Comparison with  clinical results
c2="skyblue2"
col1="mediumpurple3"

levels(datPV$Group) <- c(levels(datPV$Group), "DS-")
levels(datPV$Group) <- c(levels(datPV$Group), "DS+")
datPV[datPV$Group == "N",]$Group <- "DS-"
datPV[datPV$Group == "P",]$Group <- "DS+"
~~~

```
levels(dat$Scan) <- c(levels(dat$Scan), "t0.")
levels(dat$Scan) <- c(levels(dat$Scan), "t1.")
levels(dat$Scan) <- c(levels(dat$Scan), "t2.")
dat[dat$Scan == "T0",]$Scan <- "t0."
dat[dat$Scan == "T1",]$Scan <- "t1."
dat[dat$Scan == "T2",]$Scan <- "t2."

a6<-ggplot (aes(x= Group, y=CSS0),data=datPV[datPV$Scan=="t0",])+#
  geom_boxplot(alpha=c(0.2,0.2),col=c(col1,c2),fill=c(col1,c2))+
  geom_point()+
  theme(panel.background = element_rect(fill = 'white',colour='gray'))
+theme(panel.grid.major = element_line(colour = "gray"))

b6<-ggplot (aes(x= Group, y=KB0),data=datPV[datPV$Scan=="t0",])+#
  geom_boxplot(alpha=c(0.2,0.2),col=c(col1,c2),fill=c(col1,c2))+
  geom_point()+ylab('kb')+
  theme(panel.background = element_rect(fill = 'white',colour='gray'))
+theme(panel.grid.major = element_line(colour = "gray"))

c6<-ggplot (aes(x= Group,
y=Dinamometria.Quadricipite),data=datPV[datPV$Scan=="t0",])+ylab("Dyn.(N)")
+
  geom_boxplot(alpha=c(0.2,0.2),col=c(col1,c2),fill=c(col1,c2))+
  geom_point()+
  theme(panel.background = element_rect(fill = 'white',colour='gray'))
+theme(panel.grid.major = element_line(colour = "gray"))

d6<-ggplot (aes(x= Group, y=X6MWT),data=datPV[datPV$Scan=="t0",])+#
  geom_boxplot(alpha=c(0.2,0.2),col=c(col1,c2),fill=c(col1,c2))+
  geom_point()+
  theme(panel.background = element_rect(fill = 'white',colour='gray'))
+theme(panel.grid.major = element_line(colour = "gray"))

e6<-ggplot (aes(x= Group, y=md),data=datPV[datPV$Scan=="t0",])+ylab("Dist.
(cm)")+
  geom_boxplot(alpha=c(0.2,0.2),col=c(col1,c2),fill=c(col1,c2))+
  geom_point()+
  theme(panel.background = element_rect(fill = 'white',colour='gray'))
+theme(panel.grid.major = element_line(colour = "gray"))

f6<-ggplot (aes(x= Group, y=Age0),data=datPV[datPV$Scan=="t0",])
+ylab("Age0(y)")+#
  geom_boxplot(alpha=c(0.2,0.2),col=c(col1,c2),fill=c(col1,c2))+
  geom_point()+
  theme(panel.background = element_rect(fill = 'white',colour='gray'))
+theme(panel.grid.major = element_line(colour = "gray"))
```

````
g6<-ggarrange(a6, b6, c6, d6,e6,f6, labels = c("a","b","c","d","e","f"),
          ncol = 2, nrow = 3)
ggsave(g6, file="figure6.eps", device="eps")
```
````
````
