# Supplementary material for: Dynamic magnetic resonance imaging of muscle contraction in facioscapulohumeral muscular dystrophy
Source: Sci Rep. 2022 May 4;12:7250. doi: 10.1038/s41598-022-11147-2 (PMC9068910; doi:10.1038/s41598-022-11147-2)
Supplement: Supplementary file 5 — Supplementary Information 3. [file 41598_2022_11147_MOESM5_ESM.pdf]

```
#Analysis Data FSHD-study
#This script was applied in Linux to read and elaborate data
for descriptive statistics
install.packages("effsize")
library(effsize)
install.packages(ggplot2)
library(ggplot2)
library(rmcorr)#Repeated Measures Correlation
#Change directory to the directory where the CSV file is
baseDir='/myDir/'

my_dat = paste(baseDir, "dataFSHD_vs1_wo_WeightSize.csv", sep="" )
dat = read.table(my_dat, sep=",", header=T)
str(dat)
#Reformat the data
dat$PV<-as.factor(dat$PV)#patient(0) or volunteer(1)
dat$Scan<-
factor(dat$Scan,levels=c("t0","t1","t2","t3","T0","T1","T2"))#small t
for patients, T for healthy controls
dat$Sc01<-factor(dat$Scan,levels=c("t0","t1","T0","T1"))
dat$LegSide<-as.factor(dat$LegSide)
dat$IDN<-as.factor(dat$IDN)#volunteer ID
dat$IDL <- with(dat, interaction(IDN,LegSide))#ID per single side dataset

#dat$BMI_calc<-10000*round(dat$Weight/(dat$Size^2),5)#To confirm the tables
on the excel sheet

#FSHD patients
d0<-dat[dat$Scan=="t0",]#data from time point 0-FSHD
d1<-dat[dat$Scan=="t1",]#data from time point 1-FSHD
d2<-dat[dat$Scan=="t2",]#data from time point 2-FSHD
d3<-dat[dat$Scan=="t3",]#data from time point 3-FSHD
#Healthy Controls
dT0<-dat[dat$Scan=="T0",]#data from time point 0-healthy control
dT1<-dat[dat$Scan=="T1",]#data from time point 1-healthy control
dT2<-dat[dat$Scan=="T2",]#data from time point 2-healthy control
#Supplementary table 2
cat("Single side (FSHD,HC) datasets at baseline:",nrow(d0),nrow(dT0))
cat("Single side (FSHD,HC) datasets at t1:",nrow(d1),nrow(dT1))

#Figure 2-Calculate current and strain DIFFERENCES (from the first scan)
dat_temp<-dat
dat$DiffmA<-dat$mA #Difference in mA from the first scan
dat$DiffS<-dat$S #Difference in S from the first scan

dat$Diff_pRT<-dat$pRT #Difference in pRT (build-up rate) from the first
scan
dat$Diff_nRT<-dat$nRT #Difference in nRT (release rate) from the first scan
```

```
dat$Group="group"

for(j in levels(dat$IDL)){
  #IDL:ID of volunteer-R/L Leg
  dat_temp<-dat[dat$IDL==j,]
  dat_temp$DiffmA<-dat_temp$mA
  dat_temp$DiffS<-dat_temp$S
  dat_temp[1,]$DiffmA<-0
  dat_temp[1,]$DiffS<-0

  w<-which(dat$IDL==j,arr.ind=TRUE)

  if(length(w)>=1){dat[w[1],]$DiffmA<-0}
  if(length(w)>=1){dat[w[1],]$DiffS<-0}
  if(length(w)>=1){dat[w[1],]$Diff_pRT<-0}
  if(length(w)>=1){dat[w[1],]$Diff_nRT<-0}

  for(z in 1:nrow(dat_temp)){
    if(z>1){
      dat_temp[z,]$DiffmA<-diff(dat_temp$mA,lag=1)[z-1]
      dat_temp[z,]$DiffmA<-dat_temp[1,]$mA-dat_temp[z,]$mA
      dat_temp[z,]$DiffS<-dat_temp[1,]$S-dat_temp[z,]$S

      dat[w[z],]$DiffmA<-dat[w[z],]$mA-dat[w[1],]$mA
      dat[w[z],]$DiffS<-dat[w[z],]$S-dat[w[1],]$S
      if (z==2){
      if(dat[w[z],]$DiffS<0){
        dat[w[z],]$Group<-"N"
        dat[w[1],]$Group<-"N"
      }
      else if(dat[w[z],]$DiffS>=0){
        dat[w[z],]$Group<-"P"
        dat[w[1],]$Group<-"P"
      }
      }
      if (z!=2){dat[w[z],]$Group<-dat[w[2],]$Group}

      #extra
      dat[w[z],]$Diff_pRT<-(dat[w[z],]$pRT-dat[w[1],]$pRT)
      dat[w[z],]$Diff_nRT<-(dat[w[z],]$nRT-dat[w[1],]$nRT)
    }

  }
}
#Find number of time points for every subject
#######
w<-0
indexDat<-vector(mode="numeric", length=dim(dat)[1])
for(j in levels(dat$LegSide)){
```

```
  for(i in levels(dat$IDN)){
    print(c(i,j))
    ndat<-dat[dat$IDN==i,]
    ndat2<-ndat[ndat$LegSide==j,]
    w<-which(dat$IDN==i & dat$LegSide==j, arr.ind = TRUE)
    for (f in 1:length(w)){
      indexDat[w[f]]<-nrow(ndat2)
    }
  }
}
dat$NrScans<-indexDat

#Count patients- Supplementary Table 2: Overview of the number of dynamic
datasets
cat("Number of FSHD subjects
(t0,t1,t2,t3):",nlevels(factor(d0$IDN)),nlevels(factor(d1$IDN)),nlevels(factor(d2$IDN)),[illegible]
cat("Number of HC subject
(t0,t1,t2):",nlevels(factor(dT0$IDN)),nlevels(factor(dT1$IDN)),nlevels(factor(dT2$IDN))[illegible]

#Calculate characteristics of the patient population per dataset
library(matlab)
Age_FSHD<-zeros(nlevels(dat$IDL),1)
#Size_FSHD<-zeros(nlevels(dat$IDL),1)# not on the csv file because of
anonymization
#Weight_FSHD<-zeros(nlevels(dat$IDL),1)# not on the csv file because of
anonymization
BMI_FSHD<-zeros(nlevels(dat$IDL),1)

Age_HC<-zeros(nlevels(dat$IDL),1)
#Size_HC<-zeros(nlevels(dat$IDL),1)# not on the csv file because of
anonymization
#Weight_HC<-zeros(nlevels(dat$IDL),1) #not on the csv file because of
anonymization
BMI_HC<-zeros(nlevels(dat$IDL),1)

n<-0
for(j in levels(dat$IDL)){

  n<-n+1
  td<-dat[dat$IDL==j,]
  Age_FSHD[n]<-mean(td[td$PV=="0",]$Age0)
  Age_HC[n]<-mean(td[td$PV=="1",]$Age0)
  #Size_FSHD[n]<-mean(td[td$PV=="0",]$Size)# not on the csv file because of
anonymization
  #Size_HC[n]<-mean(td[td$PV=="1",]$Size)
  #Weight_FSHD[n]<-mean(td[td$PV=="0",]$Weight)
  #Weight_HC[n]<-mean(td[td$PV=="1",]$Weight)
  #BMI_FSHD[n]<-10000*round(Weight_FSHD[n]/(Size_FSHD[n]^2),5)
```

```
  #BMI_HC[n]<-10000*round(Weight_HC[n]/(Size_HC[n]^2),5)
}
#Calculate Male and Female Population-Study Design-Page 8 Methods
d0F<-subset(d0,d0$Sex=="F")
d0M<-subset(d0,d0$Sex=="M")
#Reported at "METHODS"
cat("Female, Male patients at
baseline:",nlevels(factor(d0F$IDN)),nlevels(factor(d0M$IDN)))

dT0F<-subset(dT0,dT0$Sex=="F")
dT0M<-subset(dT0,dT0$Sex=="M")
cat("Female, Male HCs at
baseline:",nlevels(factor(dT0F$IDN)),nlevels(factor(dT0M$IDN)))

#Calculate characteristics of the FSHD group- Study Design
#Methods
round(c(mean(Age_FSHD,na.rm=TRUE),sd(Age_FSHD,na.rm=TRUE)),1)
#round(c(mean(Size_FSHD,na.rm=TRUE),sd(Size_FSHD,na.rm=TRUE)),1)
#round(c(mean(Weight_FSHD,na.rm=TRUE),sd(Weight_FSHD,na.rm=TRUE)),1)
#round(c(mean(BMI_FSHD,na.rm=TRUE),sd(BMI_FSHD,na.rm=TRUE)),1)

#Calculate characteristics of the HC group- Study Design
round(c(mean(Age_HC,na.rm=TRUE),sd(Age_HC,na.rm=TRUE)),1)
#round(c(mean(Size_HC,na.rm=TRUE),sd(Size_HC,na.rm=TRUE)),1)
#round(c(mean(Weight_HC,na.rm=TRUE),sd(Weight_HC,na.rm=TRUE)),1)
#round(c(mean(BMI_HC,na.rm=TRUE),sd(BMI_HC,na.rm=TRUE)),1)

#RESULTS section
round(c(mean(d0$CSS,na.rm=TRUE),sd(d0$CSS,na.rm=TRUE)),2)#page 12 results
round(summary(d0$CSS0))
#Counts of severity scores for FSHD patients reported in the results
r<-hist(d0$CSS)
cat("groups of CSS0",nrow (d0[d0$CSS<=2.5,]),nrow
(d0[(d0$CSS>2.5)&(d0$CSS<=3),]),nrow
(d0[(d0$CSS>3)&(d0$CSS<=3.5),]),nrow (d0[d0$CSS>3.5,]))
#Results-1st paragraph
round(c(mean(d0$KB0,na.rm=TRUE),sd(d0$KB0,na.rm=TRUE)),2)
round(c(mean(d0$Dinamometria.Quadricipite,na.rm=TRUE),sd(d0$Dinamometria.Quadricipite,na
2)
round(c(mean(d0$X6MWT,na.rm=TRUE),sd(d0$X6MWT,na.rm=TRUE)),2)

#PV stands for patient(0)-volunteer(1)
#Supplementary Table S2-Count datasets
cat("Nr of FSHD ssd
(t0,t1,t2,t3):",nrow(dat[dat$Scan=="t0",]),nrow(dat[dat$Scan=="t1",]),nrow(dat[dat$Scan=
cat("Single side datasets for patients and HCs:
",nrow(dat[dat$PV==0,]),nrow(dat[dat$PV==1,]))

#Supplementary Table S2
```

```
dat_sub_1p<-subset(dat,dat$NrScans==1)#data having only one time point
dat_sub_2p<-subset(dat,dat$NrScans==2)#data having any two time points
dat_sub_3p<-subset(dat,dat$NrScans==3)#data having three time points
dat_sub_4p<-subset(dat,dat$NrScans==4)#data having four time points
dat_sub_min2p<-subset(dat,dat$NrScans>=2)#data having any two time points
dat_sub_min3p<-subset(dat,dat$NrScans>=3)#data having any two time points

c(nrow(subset(dat,dat$T2.VL>0)),nrow(subset(dat,dat$T2.VI>0)))#data that
have qMRI available
c(nrow(subset(dat,dat$FF.VL>0)),nrow(subset(dat,dat$FF.VI>0)))

dat_sub_qMRI<-subset(dat,dat$FF.VL>0)#data that have qMRI available
#For Table 2
c(nrow(dat_sub_qMRI[dat_sub_qMRI$Scan=="t0",]),nrow(dat_sub_qMRI[dat_sub_qMRI$Scan=="t1"
c(nrow(dat_sub_qMRI[dat_sub_qMRI$Scan=="T0",]),nrow(dat_sub_qMRI[dat_sub_qMRI$Scan=="T1"

#Data with enough datapoints to compare patients and HC (Figure 3)
#also for these datasets both the 1st and 2nd time points should be
available
iPV<-vector(mode="numeric", length=dim(dat)[1])
for(j in levels(dat$IDL)){

  wt0<-which(dat$IDL==j & dat$Scan=="t0", arr.ind = TRUE)
  wt1<-which(dat$IDL==j & dat$Scan=="t1", arr.ind = TRUE)
  wT0<-which(dat$IDL==j & dat$Scan=="T0", arr.ind = TRUE)
  wT1<-which(dat$IDL==j & dat$Scan=="T1", arr.ind = TRUE)

  sumw<-length(wt0)+length(wt1)
  if (sumw >1) {
    iPV[wt0]<-1
    iPV[wt1]<-1
  }
  hc_sumw<-length(wT0)+length(wT1)
  if (hc_sumw >1) {
    iPV[wT0]<-1
    iPV[wT1]<-1
  }

}
dat$iPV<-iPV
datPV<-subset(dat,dat$iPV==1)#
datPV_P<-datPV[datPV$Group=="P",]
datPV_N<-datPV[datPV$Group=="N",]
#Figure 3
c(nrow(datPV[datPV$Scan=="t0",]),nrow(datPV[datPV$Scan=="t1",]),nrow(datPV[datPV$Scan=="
cat("Normalized strain in datasets that present INrease of strain
(t0,t1,T0,T1):",nrow(datPV_P[datPV_P$Scan=="t0",]),nrow(datPV_P[datPV_P$Scan=="t1",]),nr
cat("Normalized strain in datasets that present DEcrease of strain
(t0,t1,T0,T1):",nrow(datPV_N[datPV_N$Scan=="t0",]),nrow(datPV_N[datPV_N$Scan=="t1",]),nr
```

```
#Figure 6
cat("CSS0 of DS+ group",mean(datPV_P$CSS,na.rm = TRUE),sd(datPV_P$CSS,na.rm
= TRUE))
cat("CSS0 of DS- group",mean(datPV_N$CSS,na.rm = TRUE),sd(datPV_N$CSS,na.rm
= TRUE))

#Supplementary Table 2-vs1
#Supplementary Table 2-vs1:FSHD
cat("ln 3,Nr. of
ssd:",nrow(dat[dat$Scan=="t0",]),nrow(dat[dat$Scan=="t1",]),nrow(dat[dat$Scan=="t2",]),n
cat("1
TP:",nrow(dat_sub_1p[dat_sub_1p$Scan=="t0",]),nrow(dat_sub_1p[dat_sub_1p$Scan=="t1",]),n
cat("2
TP:",nrow(dat_sub_2p[dat_sub_2p$Scan=="t0",]),nrow(dat_sub_2p[dat_sub_2p$Scan=="t1",]),n
cat("3
TP:",nrow(dat_sub_3p[dat_sub_3p$Scan=="t0",]),nrow(dat_sub_3p[dat_sub_3p$Scan=="t1",]),n
cat("4
TP:",nrow(dat_sub_4p[dat_sub_4p$Scan=="t0",]),nrow(dat_sub_4p[dat_sub_4p$Scan=="t1",]),n
cat("min
2:",nrow(dat_sub_min2p[dat_sub_min2p$Scan=="t0",]),nrow(dat_sub_min2p[dat_sub_min2p$Scan
cat("min
3:",nrow(dat_sub_min3p[dat_sub_min3p$Scan=="t0",]),nrow(dat_sub_min3p[dat_sub_min3p$Scan

# Supplementary Table 2-vs1: Healthy Controls
c(nrow(dat[dat$Scan=="T0",]),nrow(dat[dat$Scan=="T1",]),nrow(dat[dat$Scan=="T2",]))
c(nrow(dat_sub_1p[dat_sub_1p$Scan=="T0",]),nrow(dat_sub_1p[dat_sub_1p$Scan=="T1",]),nrow
c(nrow(dat_sub_2p[dat_sub_2p$Scan=="T0",]),nrow(dat_sub_2p[dat_sub_2p$Scan=="T1",]),nrow
c(nrow(dat_sub_3p[dat_sub_3p$Scan=="T0",]),nrow(dat_sub_3p[dat_sub_3p$Scan=="T1",]),nrow
c(nrow(dat_sub_min2p[dat_sub_min2p$Scan=="T0",]),nrow(dat_sub_min2p[dat_sub_min2p$Scan==
#Median Distance of the electrodes
summary(dat[dat$PV=="0",]$md)
summary(dat[dat$PV=="1",]$md)

#Supplementary Figure legend (Visualization of settings of current)
summary(dat[dat$Scan=="t1",]$DiffmA)
summary(dat[dat$Scan=="t2",]$DiffmA)
summary(dat[dat$Scan=="t3",]$DiffmA)
summary(dat[dat$Scan=="T1",]$DiffmA)

#Normalization #Results vs Current
plot(DiffS~DiffmA,data=dat[dat$TP>0,])#subset(dat,dat$PV==0))
lm.Diff0 <- lm(DiffS ~ DiffmA, data = dat[dat$TP>0,])
abline(a = coef(lm.Diff0)[1],   b = coef(lm.Diff0)[2],    col = "blue", lty
= "dashed",lwd=2)
s<-summary(lm.Diff0)
nFactor<-s$coefficients[2]
#This factor is used for the normalization of strain for figures
```

```
abline(a = coef(lm.Diff0)[1],   b = coef(lm.Diff0)[2]*nFactor,    col =
"red", lty = "dashed",lwd=2)

plot(DiffS*nFactor~DiffmA,data=dat[dat$TP>0,])#subset(dat,dat$PV==0))
lm.Diff1 <- lm(DiffS*nFactor ~ DiffmA, data = dat[dat$TP>0,])
abline(a = coef(lm.Diff1)[1],   b = coef(lm.Diff1)[2],    col = "blue", lty
= "dashed",lwd=2)
summary(lm.Diff1)

#Figure 4-legend (Datasets for FSHD patients, including a minimum of three
time points)
dp<-dat_sub_min3p[dat_sub_min3p$Group=="P",]
dn<-dat_sub_min3p[dat_sub_min3p$Group=="N",]
cat("Nr of FSHD-Δs+ datasets
(t0,t1,t2,t3):",nrow(dp[dp$Scan=="t0",]),nrow(dp[dp$Scan=="t1",]),nrow(dp[dp$Scan=="t2",
cat("Nr of FSHD-Δs- datasets
(t0,t1,t2,t3):",nrow(dn[dn$Scan=="t0",]),nrow(dn[dn$Scan=="t1",]),nrow(dn[dn$Scan=="t2",

data2=dat_sub_min3p[dat_sub_min3p$PV=="0"&dat_sub_min3p$Group=="N",]#ds-
group
#Estimation of reduction for the abstract
median(data2[data2$TP==3,]$S)/median(data2[data2$TP==0,]$S)

# Results pRT vs Current
#ccor_pRT<-rmrmcorr(participant=get('IDN'),get('mA'),get('pRT'),dat)
#c(ccor_pRT$r,ccor_pRT$p)

#ccor_nRT<-rmrmcorr(participant=get('IDN'),get('mA'),get('nRT'),dat)
#c(ccor_nRT$r,ccor_nRT$p)

#rmcorrelation with quantitative MRI- Table 2
corr_df<-as.data.frame(cbind(vector("numeric",
12),character(12),character(12)),stringsAsFactors = FALSE)

ccor_S_T2VL<-rmcorr(participant=get('IDN'),get('S'),get('T2.VL'),dat)
corr_df[1,1]<-as.character("S_T2VL")
corr_df[1,2]<-round(ccor_S_T2VL$r,3)
corr_df[1,3]<-round(ccor_S_T2VL$p,3)
corr_df[1,4]<-sum(!is.na(dat$T2.VL))

ccor_S_T2VI<-rmcorr(participant=get('IDN'),get('S'),get('T2.VI'),dat)
corr_df[2,1]<-as.character("S_T2VI")
corr_df[2,2]<-round(ccor_S_T2VI$r,3)
corr_df[2,3]<-round(ccor_S_T2VI$p,3)
corr_df[2,4]<-sum(!is.na(dat$T2.VI))

ccor_S_FFVL<-rmcorr(participant=get('IDN'),get('S'),get('FF.VL'),dat)
corr_df[3,1]<-as.character("S_FFVL")
corr_df[3,2]<-round(ccor_S_FFVL$r,3)
```

```
corr_df[3,3]<-round(ccor_S_FFVL$p,3)
corr_df[3,4]<-sum(!is.na(dat$FF.VL))

ccor_S_FFVI<-rmcorr(participant=get('IDN'),get('S'),get('FF.VI'),dat)
corr_df[4,1]<-as.character("S_FFVI")
corr_df[4,2]<-round(ccor_S_FFVI$r,3)
corr_df[4,3]<-round(ccor_S_FFVI$p,3)
corr_df[4,4]<-sum(!is.na(dat$FF.VI))

#Correlation Coefficients
ccor_pRT_T2VL<-rmcorr(participant=get('IDN'),get('pRT'),get('T2.VL'),dat)
corr_df[5,1]<-as.character("pRT_T2VL")
corr_df[5,2]<-round(ccor_pRT_T2VL$r,3)
corr_df[5,3]<-round(ccor_pRT_T2VL$p,3)
corr_df[5,4]<-sum(!is.na(dat$T2.VL))

ccor_pRT_T2VI<-rmcorr(participant=get('IDN'),get('pRT'),get('T2.VI'),dat)
corr_df[6,1]<-as.character("pRT_T2VI")
corr_df[6,2]<-round(ccor_pRT_T2VI$r,3)
corr_df[6,3]<-round(ccor_pRT_T2VI$p,3)
corr_df[6,4]<-sum(!is.na(dat$T2.VI))

ccor_pRT_FFVL<-rmcorr(participant=get('IDN'),get('pRT'),get('FF.VL'),dat)
corr_df[7,1]<-as.character("pRT_FFVL")
corr_df[7,2]<-round(ccor_pRT_FFVL$r,3)
corr_df[7,3]<-round(ccor_pRT_FFVL$p,3)
corr_df[7,4]<-sum(!is.na(dat$FF.VL))

ccor_pRT_FFVI<-rmcorr(participant=get('IDN'),get('pRT'),get('FF.VI'),dat)
corr_df[8,1]<-as.character("pRT_FFVI")
corr_df[8,2]<-round(ccor_pRT_FFVI$r,3)
corr_df[8,3]<-round(ccor_pRT_FFVI$p,3)
corr_df[8,4]<-sum(!is.na(dat$FF.VI))

ccor_nRT_T2VL<-rmcorr(participant=get('IDN'),get('nRT'),get('T2.VL'),dat)
corr_df[9,1]<-as.character("nRT_T2VL")
corr_df[9,2]<-round(ccor_nRT_T2VL$r,3)
corr_df[9,3]<-round(ccor_nRT_T2VL$p,3)
corr_df[9,4]<-sum(!is.na(dat$T2.VL))

ccor_nRT_T2VI<-rmcorr(participant=get('IDN'),get('nRT'),get('T2.VI'),dat)
corr_df[10,1]<-as.character("nRT_T2VI")
corr_df[10,2]<-round(ccor_nRT_T2VI$r,3)
corr_df[10,3]<-round(ccor_nRT_T2VI$p,3)
corr_df[10,4]<-sum(!is.na(dat$T2.VI))

ccor_nRT_FFVL<-rmcorr(participant=get('IDN'),get('nRT'),get('FF.VL'),dat)
corr_df[11,1]<-as.character("nRT_FFVL")
corr_df[11,2]<-round(ccor_nRT_FFVL$r,3)
```

```
corr_df[11,3]<-round(ccor_nRT_FFVL$p,3)
corr_df[11,4]<-sum(!is.na(dat$FF.VL))

ccor_nRT_FFVI<-rmcorr(participant=get('IDN'),get('nRT'),get('FF.VI'),dat)
corr_df[12,1]<-as.character("nRT_FFVI")
corr_df[12,2]<-round(ccor_nRT_FFVI$r,3)
corr_df[12,3]<-round(ccor_nRT_FFVI$p,3)
corr_df[12,4]<-sum(!is.na(dat$FF.VI))
#######################
#Revision 1-Correlations, FSHD/HC only
ccor_S_T2VL_PZ<-cor.test(dat[dat$PV=="0",]$S,dat[dat$PV=="0",]
$T2.VL,use="na.or.complete")
ccor_S_T2VL_HC<-cor.test(dat[dat$PV=="1",]$S,dat[dat$PV=="1",]
$T2.VL,use="na.or.complete")

corr_df[1,5]<-round(ccor_S_T2VL_PZ$estimate,3)
corr_df[1,6]<-round(ccor_S_T2VL_PZ$p.value,3)
corr_df[1,7]<-sum(!is.na(dat[dat$PV=="0",]$T2.VL))
corr_df[1,8]<-round(ccor_S_T2VL_HC$estimate,3)
corr_df[1,9]<-round(ccor_S_T2VL_HC$p.value,3)
corr_df[1,10]<-sum(!is.na(dat[dat$PV=="1",]$T2.VL))
######FSHD only
ccor_S_T2VI_PZ<-cor.test(dat[dat$PV=="0",]$S,dat[dat$PV=="0",]
$T2.VI,use="na.or.complete")
ccor_S_T2VI_HC<-cor.test(dat[dat$PV=="1",]$S,dat[dat$PV=="1",]
$T2.VI,use="na.or.complete")
corr_df[2,5]<-round(ccor_S_T2VI_PZ$estimate,3)
corr_df[2,6]<-round(ccor_S_T2VI_PZ$p.value,3)
corr_df[2,7]<-sum(!is.na(dat[dat$PV=="0",]$T2.VI))
corr_df[2,8]<-round(ccor_S_T2VI_HC$estimate,3)
corr_df[2,9]<-round(ccor_S_T2VI_HC$p.value,3)
corr_df[2,10]<-sum(!is.na(dat[dat$PV=="1",]$T2.VI))
#########
ccor_S_FFVL_PZ<-cor.test(dat[dat$PV=="0",]$S,dat[dat$PV=="0",]
$FF.VL,use="na.or.complete")
ccor_S_FFVL_HC<-cor.test(dat[dat$PV=="1",]$S,dat[dat$PV=="1",]
$FF.VL,use="na.or.complete")
corr_df[3,5]<-round(ccor_S_FFVL_PZ$estimate,3)
corr_df[3,6]<-round(ccor_S_FFVL_PZ$p.value,3)
corr_df[3,7]<-sum(!is.na(dat[dat$PV=="0",]$FF.VL))
corr_df[3,8]<-round(ccor_S_FFVL_HC$estimate,3)
corr_df[3,9]<-round(ccor_S_FFVL_HC$p.value,3)
corr_df[3,10]<-sum(!is.na(dat[dat$PV=="1",]$FF.VL))
#################
ccor_S_FFVI_PZ<-cor.test(dat[dat$PV=="0",]$S,dat[dat$PV=="0",]
$FF.VI,use="na.or.complete")
ccor_S_FFVI_HC<-cor.test(dat[dat$PV=="1",]$S,dat[dat$PV=="1",]
$FF.VI,use="na.or.complete")
corr_df[4,5]<-round(ccor_S_FFVI_PZ$estimate,3)
```

```
corr_df[4,6]<-round(ccor_S_FFVI_PZ$p.value,3)
corr_df[4,7]<-sum(!is.na(dat[dat$PV=="0",]$FF.VI))
corr_df[4,8]<-round(ccor_S_FFVI_HC$estimate,3)
corr_df[4,9]<-round(ccor_S_FFVI_HC$p.value,3)
corr_df[4,10]<-sum(!is.na(dat[dat$PV=="1",]$FF.VI))
#Correlation Coefficients
ccor_pRT_T2VL_PZ<-cor.test(dat[dat$PV=="0",]$pRT,dat[dat$PV=="0",]
$T2.VL,use="na.or.complete")
ccor_pRT_T2VL_HC<-cor.test(dat[dat$PV=="1",]$pRT,dat[dat$PV=="1",]
$T2.VL,use="na.or.complete")
corr_df[5,5]<-round(ccor_pRT_T2VL_PZ$estimate,3)
corr_df[5,6]<-round(ccor_pRT_T2VL_PZ$p.value,3)
corr_df[5,7]<-sum(!is.na(dat[dat$PV=="0",]$T2.VL))
corr_df[5,8]<-round(ccor_pRT_T2VL_HC$estimate,3)
corr_df[5,9]<-round(ccor_pRT_T2VL_HC$p.value,3)
corr_df[5,10]<-sum(!is.na(dat[dat$PV=="1",]$T2.VL))
###
ccor_pRT_T2VI_PZ<-cor.test(dat[dat$PV=="0",]$pRT,dat[dat$PV=="0",]
$T2.VI,use="na.or.complete")
ccor_pRT_T2VI_HC<-cor.test(dat[dat$PV=="1",]$pRT,dat[dat$PV=="1",]
$T2.VI,use="na.or.complete")
corr_df[6,5]<-round(ccor_pRT_T2VI_PZ$estimate,3)
corr_df[6,6]<-round(ccor_pRT_T2VI_PZ$p.value,3)
corr_df[6,7]<-sum(!is.na(dat[dat$PV=="0",]$T2.VI))
corr_df[6,8]<-round(ccor_pRT_T2VI_HC$estimate,3)
corr_df[6,9]<-round(ccor_pRT_T2VI_HC$p.value,3)
corr_df[6,10]<-sum(!is.na(dat[dat$PV=="1",]$T2.VI))
#####
ccor_pRT_FFVL_PZ<-cor.test(dat[dat$PV=="0",]$pRT,dat[dat$PV=="0",]
$FF.VL,use="na.or.complete")
ccor_pRT_FFVL_HC<-cor.test(dat[dat$PV=="1",]$pRT,dat[dat$PV=="1",]
$FF.VL,use="na.or.complete")
corr_df[7,5]<-round(ccor_pRT_FFVL_PZ$estimate,3)
corr_df[7,6]<-round(ccor_pRT_FFVL_PZ$p.value,3)
corr_df[7,7]<-sum(!is.na(dat[dat$PV=="0",]$FF.VL))
corr_df[7,8]<-round(ccor_pRT_FFVL_HC$estimate,3)
corr_df[7,9]<-round(ccor_pRT_FFVL_HC$p.value,3)
corr_df[7,10]<-sum(!is.na(dat[dat$PV=="1",]$FF.VL))
##
ccor_pRT_FFVI_PZ<-cor.test(dat[dat$PV=="0",]$pRT,dat[dat$PV=="0",]
$FF.VI,use="na.or.complete")
ccor_pRT_FFVI_HC<-cor.test(dat[dat$PV=="1",]$pRT,dat[dat$PV=="1",]
$FF.VI,use="na.or.complete")
corr_df[8,5]<-round(ccor_pRT_FFVI_PZ$estimate,3)
corr_df[8,6]<-round(ccor_pRT_FFVI_PZ$p.value,3)
corr_df[8,7]<-sum(!is.na(dat[dat$PV=="0",]$FF.VI))
corr_df[8,8]<-round(ccor_pRT_FFVI_HC$estimate,3)
corr_df[8,9]<-round(ccor_pRT_FFVI_HC$p.value,3)
corr_df[8,10]<-sum(!is.na(dat[dat$PV=="1",]$FF.VI))
```

```
########
ccor_nRT_T2VL_PZ<-cor.test(dat[dat$PV=="0",]$nRT,dat[dat$PV=="0",]
$T2.VL,use="na.or.complete")
ccor_nRT_T2VL_HC<-cor.test(dat[dat$PV=="1",]$nRT,dat[dat$PV=="1",]
$T2.VL,use="na.or.complete")
corr_df[9,5]<-round(ccor_nRT_T2VL_PZ$estimate,3)
corr_df[9,6]<-round(ccor_nRT_T2VL_PZ$p.value,3)
corr_df[9,7]<-sum(!is.na(dat[dat$PV=="0",]$T2.VL))
corr_df[9,8]<-round(ccor_nRT_T2VL_HC$estimate,3)
corr_df[9,9]<-round(ccor_nRT_T2VL_HC$p.value,3)
corr_df[9,10]<-sum(!is.na(dat[dat$PV=="1",]$T2.VL))
##########
ccor_nRT_T2VI_PZ<-cor.test(dat[dat$PV=="0",]$nRT,dat[dat$PV=="0",]
$T2.VI,use="na.or.complete")
ccor_nRT_T2VI_HC<-cor.test(dat[dat$PV=="1",]$nRT,dat[dat$PV=="1",]
$T2.VI,use="na.or.complete")
corr_df[10,5]<-round(ccor_nRT_T2VI_PZ$estimate,3)
corr_df[10,6]<-round(ccor_nRT_T2VI_PZ$p.value,3)
corr_df[10,7]<-sum(!is.na(dat[dat$PV=="0",]$T2.VI))
corr_df[10,8]<-round(ccor_nRT_T2VI_HC$estimate,3)
corr_df[10,9]<-round(ccor_nRT_T2VI_HC$p.value,3)
corr_df[10,10]<-sum(!is.na(dat[dat$PV=="1",]$T2.VI))
#########
ccor_nRT_FFVL_PZ<-cor.test(dat[dat$PV=="0",]$nRT,dat[dat$PV=="0",]
$FF.VL,use="na.or.complete")
ccor_nRT_FFVL_HC<-cor.test(dat[dat$PV=="1",]$nRT,dat[dat$PV=="1",]
$FF.VL,use="na.or.complete")
corr_df[11,5]<-round(ccor_nRT_FFVL_PZ$estimate,3)
corr_df[11,6]<-round(ccor_nRT_FFVL_PZ$p.value,3)
corr_df[11,7]<-sum(!is.na(dat[dat$PV=="0",]$FF.VL))
corr_df[11,8]<-round(ccor_nRT_FFVL_HC$estimate,3)
corr_df[11,9]<-round(ccor_nRT_FFVL_HC$p.value,3)
corr_df[11,10]<-sum(!is.na(dat[dat$PV=="1",]$FF.VL))
######
ccor_nRT_FFVI_PZ<-cor.test(dat[dat$PV=="0",]$nRT,dat[dat$PV=="0",]
$FF.VI,use="na.or.complete")
ccor_nRT_FFVI_HC<-cor.test(dat[dat$PV=="1",]$nRT,dat[dat$PV=="1",]
$FF.VI,use="na.or.complete")
corr_df[12,5]<-round(ccor_nRT_FFVI_PZ$estimate,3)
corr_df[12,6]<-round(ccor_nRT_FFVI_PZ$p.value,3)
corr_df[12,7]<-sum(!is.na(dat[dat$PV=="0",]$FF.VI))
corr_df[12,8]<-round(ccor_nRT_FFVI_HC$estimate,3)
corr_df[12,9]<-round(ccor_nRT_FFVI_HC$p.value,3)
corr_df[12,10]<-sum(!is.na(dat[dat$PV=="1",]$FF.VI))
colnames(corr_df)<-
c("parameters","rAll","p","nr","rFSHD","p","nr","rHC","p","nr")
write.csv(corr_df, "correlations_qMRI_strain.csv")

#Summary of T2 and FF
```

```
cat("T2_VL in FSHD:",round(c(mean(dat[dat$PV==0,]$T2.VL,na.rm =
TRUE),sd(dat[dat$PV==0,]$T2.VL,na.rm = TRUE)),2))
cat("T2 VL in HC: ",round(c(mean(dat[dat$PV==1,]$T2.VL,na.rm =
TRUE),sd(dat[dat$PV==1,]$T2.VL,na.rm = TRUE)),2))

cat("T2.VI in FSHD:",round(c(mean(dat[dat$PV==0,]$T2.VI,na.rm =
TRUE),sd(dat[dat$PV==0,]$T2.VI,na.rm = TRUE)),2))
cat("T2.VI in HC:",round(c(mean(dat[dat$PV==1,]$T2.VI,na.rm =
TRUE),sd(dat[dat$PV==1,]$T2.VI,na.rm = TRUE)),2))

cat("FF.VL in FSHD:",round(c(mean(dat[dat$PV==0,]$FF.VL,na.rm =
TRUE),sd(dat[dat$PV==0,]$FF.VL,na.rm = TRUE)),2))
cat("FF.VL in HC:",round(c(mean(dat[dat$PV==1,]$FF.VL,na.rm =
TRUE),sd(dat[dat$PV==1,]$FF.VL,na.rm = TRUE)),2))

cat("FF.VI in FSHD",round(c(mean(dat[dat$PV==0,]$FF.VI,na.rm =
TRUE),sd(dat[dat$PV==0,]$FF.VI,na.rm = TRUE)),2))
cat("FF.VI in HC",round(c(mean(dat[dat$PV==1,]$FF.VI,na.rm =
TRUE),sd(dat[dat$PV==1,]$FF.VI,na.rm = TRUE)),2))

#Analyze Groups versus physiological measurements
#Dynamometry all groups
#plot(d0$S,d0$Dinamometria.Quadricipite,ylab="Dyn",xlab="S",col=2)
plot(d0$S,d0$CSS)
#plot(d0$S~d0$X6MWT)
#plot(dat$BMI,dat$nS,col=as.factor(dat$PV))
hist(d0$CSS)
#Extra
library(ggplot2)
ggplot (aes(x= PV, y=Age0,col=2),data=dat)+xlab("FSHD/ Healthy Control")+
  geom_point()+ geom_boxplot(alpha=c(0.2,0.2),col=c(2,4),fill=c(2,4))+
  ggtitle("Age")

#TEST of populations
#Supplementary Table 1
df_p_population<-as.data.frame(cbind(vector("numeric",3),vector("numeric",
3),vector("numeric",3),vector("numeric",3),vector("numeric",
3),vector("numeric",3),vector("numeric",3),vector("numeric",
3),vector("numeric",3),vector("numeric",3)),stringsAsFactors = FALSE)

#Test the difference of FSHD patients and HCs
res1.1<-t.test(log(d0$Age0),log(dT0$Age0), var.equal=FALSE,
paired=FALSE,alternative="two.sided")
effSZ1.1<-cohen.d(log(d0$Age0),log(dT0$Age0))
dM1.1<-median(d0$Age0)-median(dT0$Age0)

res1.2<-t.test(log(d0$BMI),log(dT0$BMI), var.equal=FALSE,
paired=FALSE,alternative="two.sided")
effSZ1.2<-cohen.d(log(d0$BMI),log(dT0$BMI))
```

```
dM1.2<-median(d0$BMI)-median(dT0$BMI)

#res1.3<-t.test(log(d0$Weight),log(dT0$Weight), var.equal=FALSE,
paired=FALSE,alternative="two.sided")
#effSZ1.3<-cohen.d(log(d0$Weight),log(dT0$Weight))
#dM1.3<-median(d0$Weight)-median(dT0$Weight)

#res1.4<-t.test(log(d0$Size),log(dT0$Size), var.equal=FALSE,
paired=FALSE,alternative="two.sided")
#effSZ1.4<-cohen.d(log(d0$Size),log(dT0$Size))
#dM1.4<-median(d0$Size)-median(dT0$Size)

res1.5<-t.test(log(d0$md),log(dT0$md), var.equal=FALSE,
paired=FALSE,alternative="two.sided")
effSZ1.5<-cohen.d(log(d0$md),log(dT0$md))
dM1.5<-median(d0$md)-median(dT0$md)

res2.1<-t.test(log(d1$Age0),log(dT1$Age0), var.equal=FALSE,
paired=FALSE,alternative="two.sided")
effSZ2.1<-cohen.d(log(d1$Age0),log(dT1$Age0))
dM2.1<-median(d1$Age0)-median(dT1$Age0)

res2.2<-t.test(log(d1$BMI),log(dT1$BMI), var.equal=FALSE,
paired=FALSE,alternative="two.sided")
effSZ2.2<-cohen.d(log(d1$BMI),log(dT1$BMI))
dM2.2<-median(d1$BMI)-median(dT0$BMI)

#res2.3<-t.test(log(d1$Weight),log(dT1$Weight), var.equal=FALSE,
paired=FALSE,alternative="two.sided")
#effSZ2.3<-cohen.d(log(d1$Weight),log(dT1$Weight))
#dM2.3<-median(d1$Weight)-median(d1$Weight)

#res2.4<-t.test(log(d1$Size),log(dT1$Size), var.equal=FALSE,
paired=FALSE,alternative="two.sided")
#effSZ2.4<-cohen.d(log(d1$Size),log(dT1$Size))
#dM2.4<-median(d1$Size)-median(dT1$Size)

res2.5<-t.test(log(d1$md),log(dT1$md), var.equal=FALSE,
paired=FALSE,alternative="two.sided")
effSZ2.5<-cohen.d(log(d1$md),log(dT1$md))
dM2.5<-median(d1$md)-median(dT1$md)
#Test the difference of FSHD patients and HCs

library(effsize)
res3.1<-t.test(log(datPV[datPV$PV=="0",]$Age0),log(datPV[datPV$PV=="1",]
$Age0), var.equal=FALSE, paired=FALSE,alternative="two.sided")
effSZ3.1<- cohen.d(log(datPV[datPV$PV=="0",]
$Age0),log(datPV[datPV$PV=="1",]$Age0))
```

```
dM3.1<-median(datPV[datPV$PV=="0",]$Age0)-median(datPV[datPV$PV=="1",]
$Age0)

res3.2<-t.test(log(datPV[datPV$PV=="0",]$BMI),log(datPV[datPV$PV=="1",]
$BMI), var.equal=FALSE, paired=FALSE,alternative="two.sided")
effSZ3.2<-cohen.d(log(datPV[datPV$PV=="0",]$BMI),log(datPV[datPV$PV=="1",]
$BMI))
dM3.2<-median(datPV[datPV$PV=="0",]$BMI)-median(datPV[datPV$PV=="1",]$BMI)

#res3.3<-t.test(log(datPV[datPV$PV=="0",]$Weight),log(datPV[datPV$PV=="1",]
$Weight), var.equal=FALSE, paired=FALSE,alternative="two.sided")
#effSZ3.3<-cohen.d(log(datPV[datPV$PV=="0",]
$Weight),log(datPV[datPV$PV=="1",]$Weight))
#dM3.3<-median(datPV[datPV$PV=="0",]$Weight)-median(datPV[datPV$PV=="1",]
$Weight)

#res3.4<-t.test(log(datPV[datPV$PV=="0",]$Size),log(datPV[datPV$PV=="1",]
$Size), var.equal=FALSE, paired=FALSE,alternative="two.sided")
#effSZ3.4<-cohen.d(log(datPV[datPV$PV=="0",]
$Size),log(datPV[datPV$PV=="1",]$Size))
#dM3.4<-median(datPV[datPV$PV=="0",]$Size)-median(datPV[datPV$PV=="1",]
$Size)

res3.5<-t.test(log(datPV[datPV$PV=="0",]$md),log(dat[datPV$PV=="1",]$md),
var.equal=FALSE, paired=FALSE,alternative="two.sided")
effSZ3.5<-cohen.d(log(datPV[datPV$PV=="0",]$md),log(dat[datPV$PV=="1",]
$md))
dM3.5<-median(datPV[datPV$PV=="0",]$md)-median(datPV[datPV$PV=="1",]$md)

df_p_population[1,1]<-round(res1.1$p.value,4)
df_p_population[1,2]<-round(effSZ1.1$estimate,3)
df_p_population[1,3]<-round(dM1.1,2)
df_p_population[1,4]<-round(res1.2$p.value,4)
df_p_population[1,5]<-round(effSZ1.2$estimate,3)
df_p_population[1,6]<-round(dM1.2,2)
df_p_population[1,7]<-0#round(res1.3$p.value,4)
df_p_population[1,8]<-0#round(effSZ1.3$estimate,3)
df_p_population[1,9]<-0#round(dM1.3,2)
df_p_population[1,10]<-0#round(res1.4$p.value,4)
df_p_population[1,11]<-0#round(effSZ1.4$estimate,3)
df_p_population[1,12]<-0#round(dM1.4,2)
df_p_population[1,13]<-round(res1.5$p.value,4)
df_p_population[1,14]<-round(effSZ1.5$estimate,3)
df_p_population[1,15]<-round(dM1.5,2)

df_p_population[2,1]<-round(res2.1$p.value,4)
df_p_population[2,2]<-round(effSZ2.1$estimate,3)
df_p_population[2,3]<-round(dM2.1,2)
df_p_population[2,4]<-round(res2.2$p.value,4)
```

```
df_p_population[2,5]<-round(effSZ2.2$estimate,3)
df_p_population[2,6]<-round(dM2.2,2)
df_p_population[2,7]<-0#round(res2.3$p.value,4)
df_p_population[2,8]<-0#round(effSZ2.3$estimate,3)
df_p_population[2,9]<-0#round(dM2.3,2)
df_p_population[2,10]<-0#round(res2.4$p.value,4)
df_p_population[2,11]<-0#round(effSZ2.4$estimate,3)
df_p_population[2,12]<-0#round(dM2.4)
df_p_population[2,13]<-round(res2.5$p.value,4)
df_p_population[2,14]<-round(effSZ2.5$estimate,3)
df_p_population[2,15]<-round(dM2.5,2)

df_p_population[3,1]<-round(res3.1$p.value,4)
df_p_population[3,2]<-round(effSZ3.1$estimate,3)
df_p_population[3,3]<-round(dM3.1,2)
df_p_population[3,4]<-round(res3.2$p.value,4)
df_p_population[3,5]<-round(effSZ3.2$estimate,3)
df_p_population[3,6]<-round(dM3.2,2)
df_p_population[3,7]<-0#round(res3.3$p.value,4)
df_p_population[3,8]<-0#round(effSZ3.3$estimate,3)
df_p_population[3,9]<-0#round(dM3.3,2)
df_p_population[3,10]<-0#round(res3.4$p.value,4)
df_p_population[3,11]<-0#round(effSZ3.4$estimate,3)
df_p_population[3,12]<-0#round(dM3.4,2)
df_p_population[3,13]<-round(res3.5$p.value,4)
df_p_population[3,14]<-round(effSZ3.5$estimate,3)
df_p_population[3,15]<-round(dM3.5,2)

names(df_p_population)<-
c("Age","esz","difMed","BMI","esz","difMed","Weight","esz","difMed","Size","esz","difMed
#Supplementary Table 1
write.csv(df_p_population, "pvaluesPopulation.csv")

###Calculate characteristic values for Figure 2-legend of Figure 2
#Figure 2a
s1<-summary(nFactor*dat[dat$PV == "0" & dat$TP == 0,]$S)#FSHD patients
s2<-summary(nFactor*dat[dat$PV == "1" & dat$TP == 0,]$S)#controls
cat("Normalized strain(t0) at baseline for FSHD patients/ HC (25%,med,75%)
=", round(s1[2],5),"/",round(s2[2],5),",", round(s1[3],5),"/",round(s2[3],
5),",",round(s1[5],5),"/",round(s2[5],5))
#Figure 2b
s3<-summary(nFactor*dat[dat$PV == "0" & dat$TP == 1,]$S)#FSHD patients
s4<-summary(nFactor*dat[dat$PV == "1" & dat$TP == 1,]$S)#controls
cat("Normalized strain(t1) for FSHD patients/ HC (25%,med,75%) =",
round(s3[2],5),"/",round(s4[2],5),",",round(s3[3],5),"/",round(s4[3],
5),",",round(s3[5],5),"/",round(s4[5],5))

#Figure 2c
s5<-summary(dat[dat$PV == "0" & dat$TP == 0,]$mA)#FSHD patients
```

```
s6<-summary(dat[dat$PV == "1" & dat$TP == 0,]$mA)#controls
cat("Current applied in mA at baseline for FSHD patients/ HC (25% quantile)
=", round(s5[2],1),"/",round(s6[2],1))
cat("Current applied in mA at baseline for FSHD patients/ HC (median) =",
round(s5[3],1),"/",round(s6[3],1))
cat("Current applied in mA at baseline for FSHD patients/ HC (75% quantile)
=", round(s5[5],1),"/",round(s6[5],1))
#Figure 2d
s7<-summary(dat[dat$PV == "0" & dat$TP == 1,]$mA)#FSHD patients
s8<-summary(dat[dat$PV == "1" & dat$TP == 1,]$mA)#controls
cat("Current applied in mA at t1 for FSHD patients/ HC (25% quantile) =",
round(s7[2],1),"/",round(s8[2],1))
cat("Current applied in mA at t1 for FSHD patients/ HC (median) =",
round(s7[3],1),"/",round(s8[3],1))
cat("Current applied in mA at t1 for FSHD patients/ HC (75% quantile) =",
round(s7[5],1),"/",round(s8[5],1))

#Figure 2e
s9<-summary(dat[dat$PV == "0" & dat$TP == 0,]$pRT)#FSHD patients
s10<-summary(dat[dat$PV == "1" & dat$TP == 0,]$pRT)#HC patients
cat("pRT at t0 for FSHD patients/ HC (25%,med,75%) =", round(s9[2],
2),"/",round(s10[2],2),",",round(s9[3],2),"/",round(s10[3],
2),",",round(s9[5],2),"/",round(s10[5],2))
#Figure 2f
s11<-summary(dat[dat$PV == "0" & dat$TP == 1,]$pRT)#FSHD patients
s12<-summary(dat[dat$PV == "1" & dat$TP == 1,]$pRT)#HC patients
cat("pRT at t1 for FSHD/ HC (25%,med,75%) =", round(s11[2],
2),"/",round(s12[2],2),",",round(s11[3],2),"/",round(s12[3],
2),",",round(s11[5],2),"/",round(s12[5],2))

#Figure 2g
s13<-summary(dat[dat$PV == "0" & dat$TP == 0,]$nRT)#FSHD patients
s14<-summary(dat[dat$PV == "1" & dat$TP == 0,]$nRT)#HC patients
cat("nRT at t0 for FSHD patients/ HC (25% quantile) =", round(s13[2],
2),"/",round(s14[2],2),",", round(s13[3],2),"/",round(s14[3],2),",",
round(s13[5],2),"/",round(s14[5],2))

#Figure 2h
s15<-summary(dat[dat$PV == "0" & dat$TP == 1,]$nRT)#FSHD patients
s16<-summary(dat[dat$PV == "1" & dat$TP == 1,]$nRT)#HC patients
cat("nRT at t1 for FSHD patients/ HC (25% quantile) =", round(s15[2],
2),"/",round(s16[2],2),",", round(s15[3],2),"/",round(s16[3],2),",",
round(s15[5],2),"/",round(s16[5],2))

#Figure 6 Correlations
corr_extra<-as.data.frame(cbind(vector("numeric",
3),character(3),character(3)),stringsAsFactors = FALSE)
colnames(corr_extra)<-c("parameters","r","p")
```

```
#Results page 14- "correlation coefficients of strain "
corr_DQ<-
rmcorr(participant=get('IDN'),get('S'),get('Dinamometria.Quadricipite'),d0)
corr_CS<-rmcorr(participant=get('IDN'),get('S'),get('CSS0'),d0)
corr_WT<-rmcorr(participant=get('IDN'),get('S'),get('X6MWT'),d0)
corr_KB0<-rmcorr(participant=get('IDN'),get('S'),get('KB0'),d0)

corr_extra[1,1]<-as.character("S_DynQ")
corr_extra[1,2]<-round(corr_DQ$r,3)
corr_extra[1,3]<-round(corr_DQ$p,3)

corr_extra[2,1]<-as.character("S_KB0")
corr_extra[2,2]<-round(corr_KB0$r,3)
corr_extra[2,3]<-round(corr_KB0$p,3)

corr_extra[3,1]<-as.character("S_CSS")
corr_extra[3,2]<-round(corr_CS$r,3)
corr_extra[3,3]<-round(corr_CS$p,3)

corr_extra[4,1]<-as.character("S_X6MWT")
corr_extra[4,2]<-round(corr_WT$r,3)
corr_extra[4,3]<-round(corr_WT$p,3)
write.csv(corr_extra, "corr_S_Population.csv")
```
